# Proximal Policy Optimization-guided novelty-aware multi-strategy particle swarm optimization for adaptive traffic signal optimization

**DOI:** 10.3389/frai.2026.1965356

**Published:** 2026-10-23

**Authors:** Manikandan R., Arun Pandian J.

**Affiliations:** 1 School of Computer Science Engineering and Information Systems, Vellore Institute of Technology, Vellore, Tamil Nadu, India; 2 Centre for Artificial Intelligence Research, Vellore Institute of Technology, Vellore, Tamil Nadu, India

**Keywords:** adaptive traffic control, intelligent transportation systems, OpenStreetMap (OSM), Proximal Policy Optimization (PPO), SUMO, traffic signal optimization

## Abstract

**Introduction:**

Traffic signal timing is a non-linear, stochastic and time-varying optimization problem. Particle Swarm Optimization (PSO), Genetic Algorithm (GA) and Ant Colony Optimization (ACO) run one fixed search strategy for the whole run, which often leads to early convergence on a poor timing plan. This study proposes PPO-NAMSPSO, which changes the search strategy during the run.

**Methods:**

A Proximal Policy Optimization (PPO) agent observes the state of a Novelty-Aware Multi-Strategy PSO (NAMSPSO) and, at each iteration, selects one of six operators: standard PSO update only, novelty-guided exploration, elite-guided update, mutation, restart, or local search. Candidates are ranked on waiting time, queue length and behavioral novelty. An elite pool guides the swarm, and stagnation recovery reinitializes the worst half of the particles after four iterations without improvement. We tested the framework in the Simulation of Urban Mobility (SUMO) on a synthetic 3 × 3 grid and on an OpenStreetMap-derived network with 25 signalized intersections, at medium and high demand. PSO, GA, ACO and fixed-time control were compared, and each optimizer was tuned with Optuna under the same budget. Each optimizer was trained with five seeds and evaluated over 100 runs.

**Results:**

Against PSO, average waiting time fell from 105.52 s to 96.97 s (8.10%) on the grid and from 48.88 s to 33.07 s (32.35%) on the OSM network, where its standard deviation fell from 17.42 s to 3.54 s. Under high demand on the grid, waiting time was 207.72 s against 315.91 s for PSO. PPO-NAMSPSO had the lowest waiting time of the five methods in all three reported comparisons. On the OSM network, ANOVA and Kruskal-Wallis tests found differences among methods (*p* < 0.001), and Holm-corrected Welch tests against each method gave *p* ≤ 1.17 × 10^−5^, with Cohen's d from 0.71 to 2.33. Removing the PPO agent raised mean waiting time from 60.38 s to 64.91 s under high demand.

**Discussion:**

The gain was larger on the OSM network than on the grid. The agent used all six operators, and the elite-guided update was chosen most often (20.61% of selections). Demand was synthetic, so validation on field data remains open.

## 1 Introduction

Traffic congestion is becoming a major issue in many urban transportation systems because of rapid urbanization and the increasing number of vehicles on roads (Gordon and Newell, 1983; Hunt et al., 1981). As vehicle density increases, urban road networks become heavily congested, leading to travel delay, long queues at intersections, fuel consumption and higher carbon dioxide (*CO*_2_) emissions (Papageorgiou et al., 1991). These problems not only affect the traffic flow but also cause environmental and economic problems in urban areas. Traditionally, traffic movement at intersections was controlled using fixed traffic signal systems with predefined timing plans.

Traffic signal control is one of the most widely adopted approaches for managing traffic flow at urban intersections. Earlier systems mainly depended on fixed-time traffic signal control, where signal timings were determined using historical traffic data and operated using predefined phase sequences. Methods such as Webster's method are generally used to calculate signal cycle length and green time allocation (Webster, 1958). Although fixed-time systems are simple to implement, they are not suitable for dynamic traffic environments because traffic demand changes continuously throughout the day. During peak traffic periods, fixed signal timings often lead to inefficient traffic flow and increased waiting time.

Traffic conditions at intersections are not constant throughout the day. Hence, to make traffic control more flexible, actuated traffic signal systems were developed. These systems utilize data obtained from sensors (for example, loop detectors) and adapt their signal timings to actual traffic conditions (Liu et al., 2005). Compared to fixed-time systems, actuated systems improve traffic flow by responding to changing traffic demand. However, they are usually restricted to individual intersections and fail to coordinate traffic over a wider urban network. To overcome these limitations, adaptive traffic signal control systems such as Split Cycle Offset Optimization Technique (SCOOT) and Sydney Coordinated Adaptive Traffic System (SCATS) were developed. These systems use real-time traffic information from several intersections to optimize cycle length, phase split, and signal offsets in the urban traffic network (Hunt et al., 1981; Sims and Dobinson, 1980). Adaptive systems improve traffic efficiency, but they require costly sensing infrastructure, complicated calibration methods and high implementation costs. They also rely mainly on pre-defined optimization rules instead of adaptive learning behavior in their decision-making process.

To overcome these limitations, optimization-based traffic signal control approaches have been developed (Celtek et al., 2020). These methods optimize signal parameters such as cycle length, green split allocation, and phase sequence to enhance traffic performance. The objective of traffic signal optimization is to minimize average waiting time, queue length, travel delay, fuel consumption, and emissions. Particle Swarm Optimization (PSO), due to its simple implementation, low computational complexity and fast convergence behavior, has become one of the most widely used optimization techniques (Kennedy and Eberhart, 1995). PSO has shown promising performance in solving non-linear traffic signal optimization problems. However, conventional PSO still has some limitations, including premature convergence, limited exploration capability and diversity reduction of the swarm. These issues reduce optimization performance under highly dynamic and congested traffic conditions.

Several advanced PSO variants, including Multi-Objective Particle Swarm Optimization (MOPSO), have been proposed to improve optimization performance. MOPSO techniques generate multiple Pareto-optimal solutions that can balance multiple conflicting objectives simultaneously (Coello Coello et al., 2004). Consequently, MOPSO is well-suited for traffic signal optimization problems where several traffic performance measures need to be optimized together. Although existing MOPSO-based methods have shown promising performance, most of these methods still rely on fixed or linearly varying parameters and fail to adapt swarm behavior according to the optimization process. Consequently, maintaining a suitable balance between exploration and exploitation in a dynamic traffic environment remains a significant challenge.

Numerous studies have investigated traffic signal optimization using PSO and MOPSO techniques, and some enhancements have been reported in reducing traffic delay and congestion. However, several limitations remain in existing methods. Many existing approaches continue to use fixed parameter settings or simple tuning strategies, which compromise their adaptability under dynamic traffic conditions. The optimization process often converges too early, and the diversity of the swarm is lost, particularly in high traffic density situations. Furthermore, most existing approaches rely primarily on fitness-based particle movement and do not incorporate novelty-driven exploration mechanisms to encourage the search for unexplored solution regions. As a result, particles may become trapped in local optima, reducing optimization effectiveness under complex traffic conditions. In addition, dedicated stagnation recovery mechanisms are rarely considered, limiting the ability of the swarm to restore exploration capability once convergence begins. Previous studies have primarily focused on improving optimization algorithms and their applications. Although several PSO variants have been proposed, most employ a single particle update mechanism throughout the optimization process. Consequently, they cannot dynamically switch among complementary search operators according to the current optimization state, leading to an ineffective balance between exploration and exploitation. Furthermore, limited attention has been given to integrating adaptive optimization strategy selection with novelty-aware exploration, elite archive preservation, and stagnation recovery within a unified optimization framework. Table 1 summarizes the optimization algorithms used for comparative evaluation.

**Table 1:** Summary of optimization algorithms used for comparative evaluation.

Algorithm	Optimization principle	Main advantages	Main limitations	Reason for inclusion
PSO	Swarm-based population optimization using velocity and position updates	Fast convergence and low computational complexity	Premature convergence and loss of population diversity	Baseline swarm optimization method
GA	Evolutionary optimization based on selection, crossover, and mutation	Strong global search capability	Slow convergence and sensitivity to parameter settings	Widely used evolutionary optimization method
ACO	Pheromone-based swarm intelligence optimization	Effective path exploration and adaptive search	Slower convergence under complex optimization conditions	Representative swarm intelligence algorithm
PPO-NAMSPSO	PPO-guided novelty-aware multi-strategy particle swarm optimization	Adaptive strategy selection, novelty-aware exploration, elite archive preservation, and stagnation recovery	Designed to overcome the limitations of existing optimization methods	Proposed method

Despite the recent progress in reinforcement learning-based traffic signal control, existing approaches primarily employ learned policies to directly control traffic signal phases or timing decisions. In contrast, the proposed framework uses reinforcement learning at the meta-optimization level to dynamically select complementary search operators within a population-based optimization process. Therefore, the novelty of this work does not arise from PPO, PSO, or novelty mechanisms individually, but from their task-specific integration, where PPO functions as a meta-level adaptive operator-selection mechanism within Policy Optimization-guided Novelty-Aware Multi-Strategy Particle Swarm Optimization(PPO-NAMSPSO) for adaptive traffic signal optimization.

To address these limitations, this paper proposes the PPO-NAMSPSO framework for adaptive traffic signal optimization. The proposed framework integrates reinforcement learning with novelty-aware multi-strategy particle swarm optimization and dynamically selects the most appropriate optimization strategy from six complementary search operators according to the current optimization state. This design aims to improve the balance between exploration and exploitation while preserving swarm diversity and providing recovery from optimization stagnation.

The proposed framework is implemented using SUMO and TraCI and evaluated under different traffic-demand conditions to minimize average vehicle waiting time and queue length. Experimental evaluation, comparative analysis, ablation studies, and non-parametric statistical analysis are performed to validate the effectiveness and robustness of the proposed framework.

The key findings of this study are summarized below:

1. A PPO-NAMSPSO framework for adaptive traffic signal optimization in which PPO operates at the meta-optimization level to adaptively select complementary search strategies within a novelty-aware multi-strategy particle swarm optimization process.
2. An adaptive operator-selection mechanism based on PPO, which dynamically selects one of six complementary search operators according to the current optimization state, enabling a better balance between exploration and exploitation during the optimization process.
3. A novelty-aware fitness formulation combined with an elite archive and a stagnation-recovery mechanism to maintain swarm diversity and improve optimization robustness.
4. A parallel SUMO–TraCI simulation–optimization framework for efficient microscopic traffic signal optimization.
5. Comprehensive experimental validation on both a synthetic benchmark traffic network and an OSM-derived urban traffic network, covering different traffic-demand conditions and supported by non-parametric statistical significance analysis.

The remaining sections of the paper are structured as follows. Section 2 reviews the existing literature on traffic signal optimization, including conventional control strategies, metaheuristic optimization techniques, and intelligent transportation systems. The limitations of the existing approaches and the research gaps that motivate this study are also discussed. Section 3 presents the proposed PPO-NAMSPSO framework, including the traffic signal optimization formulation, particle representation, novelty-aware fitness evaluation, elite archive, multi-strategy particle update, PPO-based adaptive optimization strategy selection, reward function, policy learning, and the overall optimization algorithm. Section 4 describes the experimental setup, simulation environment, parameter settings, evaluation metrics, comparative methods and performance analysis. The effectiveness of the proposed framework is evaluated through convergence analysis, comparative evaluation and ablation studies. Section 5 concludes the paper by summarizing the key findings, limitations, and future research directions.

## 2 Related work

Recent advances in intelligent transportation systems have significantly improved traffic signal control through optimization-based and data-driven approaches. Although fixed-time traffic signal control is computationally simple and easy to implement, it exhibits limited adaptability under dynamic traffic conditions. Consequently, fixed-time traffic signal control often results in excessive queue formation, longer waiting times, traffic congestion, increased fuel consumption and environmental pollution. To overcome these limitations, adaptive and optimization-driven traffic signal control approaches have been extensively studied (Bhattacharyya et al., 2026). Priority-based adaptive traffic signal control dynamically allocates green time according to real-time traffic information, including queue length, vehicle density, and waiting time obtained from traffic sensors. However, their dependence on local traffic measurements limits network-wide traffic coordination in large-scale urban environments. Moreover, their effectiveness depends on the sensor accuracy and the reliability of the communication (Mondal et al., 2022).

Model Predictive Control (MPC) improves traffic signal adaptability by optimizing signal timings over a finite prediction horizon. Although MPC improves predictive traffic coordination, its computational complexity increases significantly with larger traffic networks and longer prediction horizons. Moreover, repeated optimization at every control interval limits the real-time applicability of MPC approaches in large-scale urban environments (Khelafa et al., 2022). Optimization-based traffic signal control has gained considerable attention because of its ability to improve multiple traffic performance metrics simultaneously. Traffic signal optimization is an inherently multi-objective problem, requiring a trade-off between operational efficiency and environmental sustainability (Zhang et al., 2023). Stochastic simulation-based optimization frameworks have also been proposed to address the uncertainties associated with the traffic demand and vehicle arrival patterns. Although simulation-based optimization improves robustness under uncertain traffic conditions, repeated traffic simulation increases computational cost and limits real-time applicability (Zheng et al., 2023).

Metaheuristic optimization algorithms have been widely applied to traffic signal optimization because of their ability to solve highly non-linear and non-convex optimization problems. Among these methods, Genetic Algorithms (GA) have been extensively used for traffic signal optimization because of their strong global search capability. However, GA often suffers from reduced population diversity during the optimization process, leading to premature convergence in dynamic traffic environments (Kim et al., 2023). Memory-assisted GA approaches have been proposed to improve convergence by exploiting historical optimization information. However, excessive reliance on historical information reduces exploration capability and limits adaptability under dynamic traffic conditions (Kianian et al., 2025). However, integrating multiple optimization mechanisms increases computational complexity and parameter tuning difficulty (Hachim and Swadi, 2024). Consequently, these methods remain computationally expensive for real-time traffic signal control applications (Akopov and Beklaryan, 2024).

Swarm intelligence algorithms have been widely applied to traffic signal optimization because of their distributed search behavior and adaptive optimization capabilities (Liao et al., 2024; Tran et al., 2022). Algorithms such as Ant Colony Optimization (ACO), Artificial Bee Colony (ABC), and Artificial Fish Swarm Optimization (AFSO) have demonstrated competitive optimization performance in traffic signal control applications (Liao et al., 2024; Tran et al., 2022). ABC has also been applied to multi-objective traffic signal optimization (Liao et al., 2024). However, swarm-based optimization methods remain sensitive to parameter initialization and tuning, which can lead to unstable convergence and reduced optimization robustness under dynamic traffic conditions (He et al., 2024).

Particle Swarm Optimization (PSO) has been widely adopted for traffic signal optimization because of its simple implementation, low computational complexity, and fast convergence. PSO has demonstrated effective performance in reducing traffic delay and queue length in urban traffic signal control systems (Shi et al., 2021). Several advanced PSO variants, including chaotic PSO and Multi-Objective Particle Swarm Optimization (MOPSO), have been proposed to improve optimization performance. Pareto-based PSO techniques have also been developed to simultaneously optimize multiple conflicting objectives (Qin et al., 2023). MOPSO has been widely applied to traffic signal optimization to address multiple conflicting objectives simultaneously (Guo et al., 2023). These methods improve the trade-off among traffic delay, queue length, throughput, and emissions (Guo et al., 2023). However, many existing MOPSO approaches rely on static or linearly varying optimization parameters, limiting adaptability under highly dynamic traffic conditions. Consequently, these methods may experience convergence instability, reduced search diversity, and premature convergence. Adaptive optimization techniques have been proposed to improve convergence stability and optimization robustness under dynamic traffic conditions. Artificial Fish Swarm Optimization (AFSO) employs adaptive exploration strategies to improve optimization performance. However, AFSO-based approaches may still exhibit slow convergence and parameter sensitivity under highly dynamic traffic conditions (Wei and Ju, 2024). Region-based PSO techniques have been proposed to improve optimization efficiency by focusing the search within promising regions of the solution space (Zhang et al., 2022). Adaptive PSO approaches dynamically adjust optimization parameters to balance exploration and exploitation during the optimization process (Nguyen and Jung, 2021). Although these strategies improve convergence quality, parameter sensitivity and adaptive parameter control remain challenging under highly dynamic traffic environments. Urban traffic signal optimization remains a highly dynamic and non-stationary problem because traffic demand, vehicle arrival rates, and queue propagation continuously change over time. Consequently, optimization algorithms employing static parameter settings often fail to maintain robust performance under varying traffic conditions. Maintaining an effective balance between exploration and exploitation remains a major challenge. Therefore, adaptive optimization strategy selection mechanisms are required to improve convergence stability, preserve swarm diversity, and enhance optimization robustness in dynamic traffic environments (Gonçalves et al., 2023).

Recent advances in deep reinforcement learning have enabled adaptive traffic signal control through continuous interaction with dynamic traffic environments. Algorithms such as Deep Q-Network (DQN) (Wu et al., 2022), Advantage Actor-Critic (A2C) (Mo et al., 2022), and Proximal Policy Optimization (PPO) have demonstrated promising performance by learning traffic control policies directly from simulation. Pan (2023) investigated reinforcement-learning-based traffic signal control using a DQN-based approach and demonstrated the potential of learning-based control for improving traffic efficiency under varying traffic conditions. Similarly, Mon et al. (2024) developed a multi-agent deep reinforcement learning framework for traffic signal control in oversaturated urban networks, where multiple agents learn traffic signal policies based on traffic-state information. Mon et al. (2024); Oukebdane (2026) further proposed a decentralized multi-agent DRL approach for jointly controlling pedestrian and vehicle traffic signals, demonstrating the applicability of decentralized learning to heterogeneous urban traffic environments. Koch et al. (2023) investigated PPO-based adaptive traffic signal control and evaluated its effects on traffic flow and energy consumption, demonstrating the potential of PPO for dynamically controlling multiple traffic signals. PPO has attracted particular attention because of its stable policy updates, improved sample efficiency, and robust convergence (Wang et al., 2025). However, existing DRL-based traffic signal control approaches primarily employ reinforcement learning as a direct control mechanism for selecting signal phases or signal timing decisions. They generally do not exploit reinforcement learning to adapt the search behavior of population-based optimization algorithms. Consequently, an opportunity remains to integrate PPO with swarm-based optimization to dynamically select optimization strategies according to the current optimization state, thereby improving the balance between exploration and exploitation while maintaining search diversity and convergence robustness.

However, existing DRL-based traffic signal control approaches primarily employ reinforcement learning as a direct control mechanism for selecting signal phases or signal timing decisions. They generally do not exploit reinforcement learning to adapt the search behavior of population-based optimization algorithms. In particular, the dynamic selection of complementary search operators according to the current optimization state remains insufficiently explored. This limitation motivates the development of a hybrid framework in which PPO is used not as a direct traffic signal controller, but as an adaptive operator-selection mechanism within a swarm-based optimization process. The proposed PPO-NAMSPSO framework therefore combines the policy-learning capability of PPO with the global search capability of NAMSPSO to dynamically balance exploration and exploitation, while a novelty-aware fitness mechanism, elite archive, and stagnation recovery strategy are employed to preserve search diversity and improve convergence robustness.

Although significant advancements have been achieved in traffic signal optimization using evolutionary and swarm intelligence algorithms, several important limitations remain unresolved. Most existing optimization approaches rely on static or linearly varying optimization parameters, limiting their adaptability under highly dynamic traffic conditions. Furthermore, maintaining an effective balance between convergence speed, search diversity, and computational efficiency remains a major challenge in large-scale traffic optimization problems. Existing optimization approaches generally rely on predefined particle update strategies throughout the optimization process. Consequently, they cannot intelligently select different search operators as the optimization state evolves. Therefore, there is a need for an adaptive optimization framework capable of dynamically selecting the most appropriate search strategy while maintaining swarm diversity, improving convergence robustness, and avoiding premature convergence under dynamic traffic conditions.

## 3 Methodology

### 3.1 Overview

This section presents the framework of the proposed PPO-NAMSPSO for adaptive traffic signal optimization. The methodology starts with the formulation of the traffic signal optimization problem and the overall optimization framework and the standard Particle Swarm Optimization (PSO) algorithm. Subsequently, the proposed PPO-NAMSPSO algorithm, including its novelty-guided exploration, elite-guided search, mutation-based search, and local search operators, is described. The integration of Proximal Policy Optimization (PPO) for adaptive optimization strategy selection is then presented through the optimization state representation, action space, reward function, and policy learning process. Finally, the Optuna–TPE-based hyperparameter initialization, the complete hybrid PPO–NAMSPSO optimization algorithm, and the computational complexity and convergence analysis are discussed.

### 3.2 System model and problem formulation

The traffic intersection model adopted in this study is illustrated in Figure 1. Each signalized intersection consists of incoming and outgoing approaches through which vehicles enter and leave the intersection. Traffic performance is evaluated using two primary traffic characteristics, namely average vehicle waiting time and queue length, which are measured on the incoming approaches. Figure 1b illustrates the eight possible traffic movement patterns, including straight-through and turning movements, that represent vehicle movements at the intersection. Although eight traffic movement patterns are considered for modeling traffic flow, these movements are grouped into non-conflicting signal phases during traffic signal optimization.

**Figure 1:**
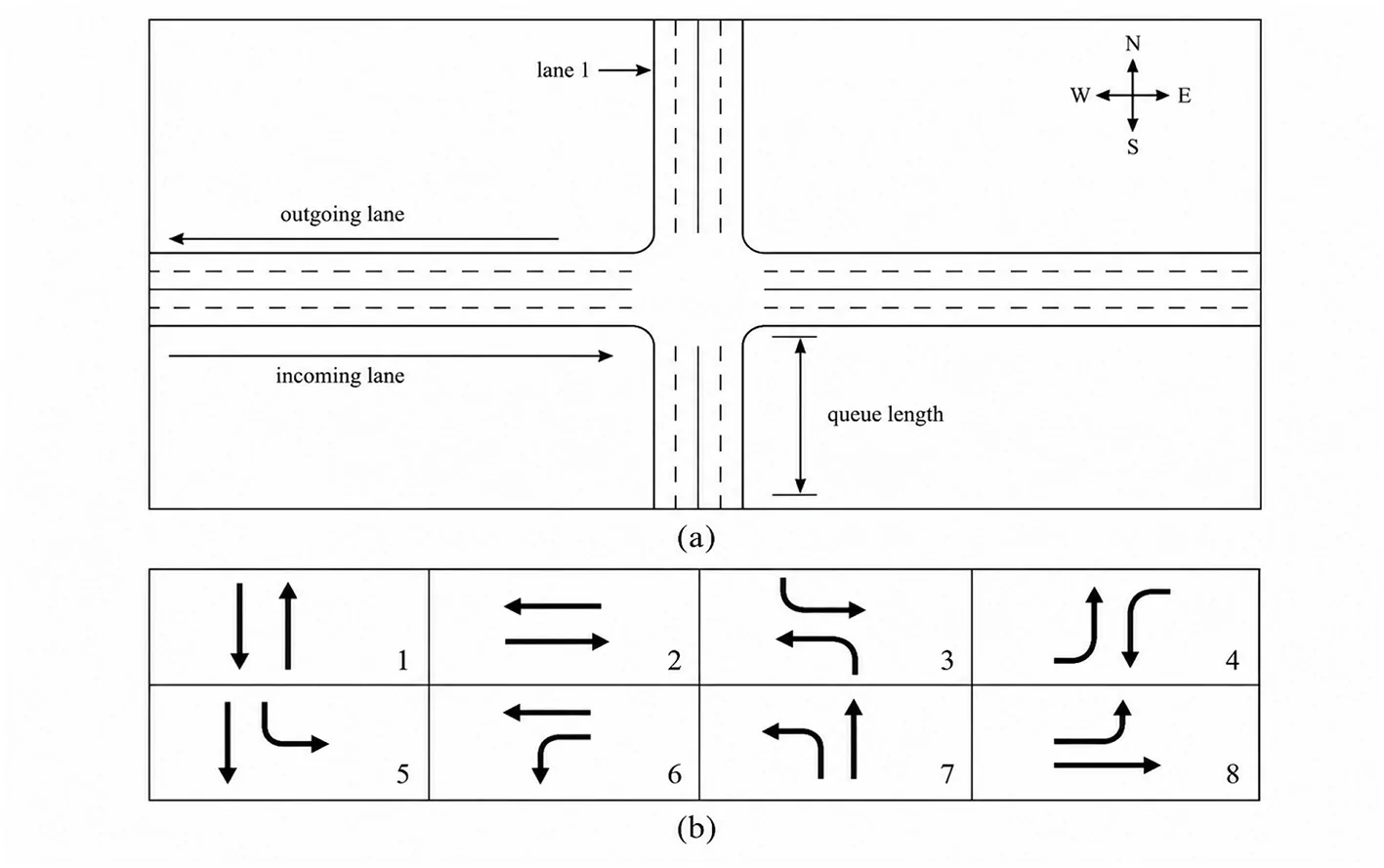
Traffic intersection model adopted in this study. **(a)** Signalized intersection showing incoming and outgoing approaches used for measuring traffic performance. **(b)** Eight possible vehicle movement patterns considered during traffic modeling. During optimization, these movements are grouped into non-conflicting signal phases for traffic signal control.

Urban traffic signal optimization is formulated as a constrained optimization problem to determine an optimal traffic signal timing configuration that reduces network congestion and improves traffic mobility. In the proposed framework, the optimization process is performed using a microscopic traffic simulation environment in which every candidate traffic signal timing plan is evaluated under identical traffic demand conditions. The optimization algorithm iteratively modifies the traffic signal parameters based on the observed traffic performance until an optimal solution is obtained.

Unlike conventional traffic signal optimization methods that directly optimize signal phase durations, the proposed framework employs a genome-based representation.

Let


$$\begin{array}{l} \text{X}=\left[x_{1},x_{2},\ldots ,x_{D}\right], \end{array}$$ (1)

denotes a candidate solution, where *D* represents the total number of optimization variables encoded in the traffic signal genome, and *x*_*d*_ denotes the *d*^th^ optimization variable. The genome dimension is determined automatically from the traffic signal configuration of the optimized network. Instead of directly representing signal timings, the genome encodes the traffic signal parameters that are subsequently decoded into cycle lengths and green signal split ratios before constructing the corresponding traffic signal configuration within the SUMO simulation environment. This representation enables the optimization algorithm to efficiently explore the search space while maintaining feasible traffic signal timing plans. The formulation is consistent with the genome decoding process implemented in the proposed optimization framework.

The decoding process is expressed as


$$\begin{array}{l} \left({\text{C}}_{L},{\text{G}}_{S}\right)=f_{\text{decode}}\left(\text{X}\right), \end{array}$$ (2)

where **C**_*L*_ denotes the decoded cycle-length vector, **G**_*S*_ denotes the decoded green-signal-split vector, and *f*_decode_(·) represents the genome decoding function that transforms the candidate genome into a feasible traffic signal timing configuration for the SUMO microscopic traffic simulator.

For every candidate solution, the decoded traffic signal configuration is executed within the microscopic traffic simulator, which returns the corresponding traffic performance measures. The optimization objective is therefore formulated as


$$\begin{array}{l} {\text{X}}^{*}=\arg\underset{\text{X}}{\min}J\left(\text{X}\right), \end{array}$$ (3)

where *J*(**X**) represents the traffic optimization objective obtained by evaluating the decoded traffic signal timing plan in the SUMO microscopic traffic simulator.

In the present study, the average vehicle waiting time is selected as the optimization objective. Accordingly, the objective function is defined as


$$\begin{array}{l} J\left(\text{X}\right)=W\left(\text{X}\right), \end{array}$$ (4)

where *W*(**X**) denotes the average vehicle waiting time obtained by evaluating the traffic signal plan encoded by **X** in the SUMO simulation.

The mathematical formulation presented in this section defines the optimization problem addressed in this study. The overall optimization framework adopted to solve this problem is described in the following section, followed by the mathematical formulation of the proposed PPO-NAMSPSO based adaptive optimization strategy selection mechanism.

### 3.3 Proposed PPO-NAMSPSO optimization framework

The proposed traffic signal optimization framework integrates microscopic traffic simulation, NAMSPSO, and PPO into a unified optimization architecture. Figure 2 illustrates the overall PPO-NAMSPSO framework for adaptive traffic signal optimization. The framework combines the global search capability of Particle Swarm Optimization with reinforcement learning-based adaptive strategy selection to improve search efficiency under dynamic traffic conditions. In contrast to conventional PSO, which employs a fixed particle update strategy throughout the optimization process, the proposed framework incorporates multiple complementary search strategies and enables the PPO agent to dynamically select the most appropriate optimization operator according to the current optimization state. This adaptive operator selection mechanism effectively balances global exploration and local exploitation, thereby improving optimization performance and accelerating convergence toward high-quality traffic signal timing plans.

**Figure 2:**
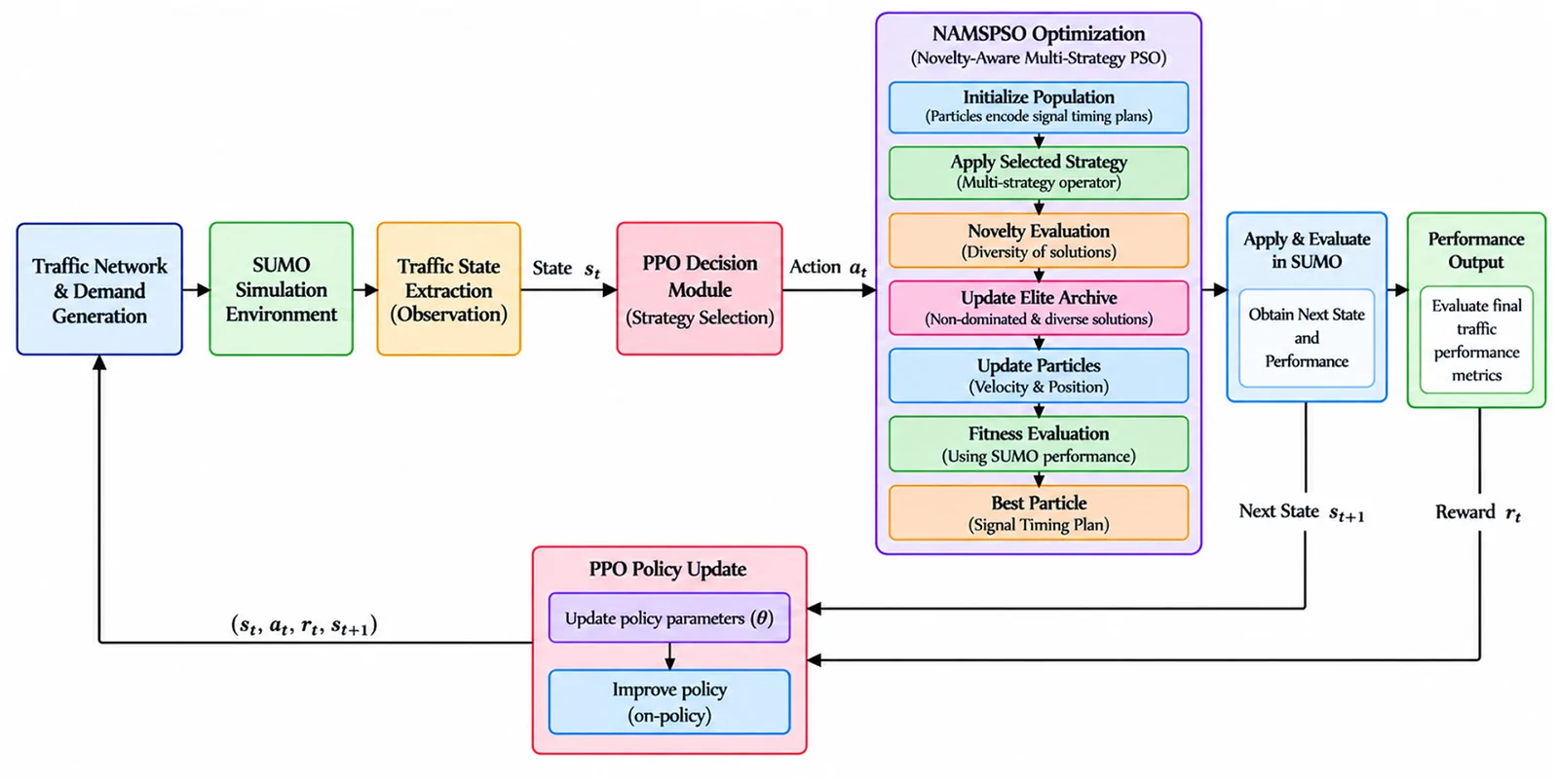
Overall PPO-NAMSPSO framework for adaptive traffic signal optimization.

### 3.4 Standard particle swarm optimization

The algorithm searches for the optimal solution by iteratively updating a population of candidate solutions, referred to as particles. Each particle adjusts its movement according to its previous search experience and the best solution identified by the swarm. During each optimization iteration, the particle velocity is updated according to its previous velocity, personal best position, and the global best solution identified by the swarm. The particle velocity is updated according to


$$\begin{array}{l} {\text{v}}_{i,k+1}=\omega {\text{v}}_{i,k}+c_{1}r_{1}\left({\text{p}}_{i,k}-{\text{x}}_{i,k}\right)+c_{2}r_{2}\left({\text{g}}_{k}-{\text{x}}_{i,k}\right), \end{array}$$ (5)

where ω denotes the inertia weight, *c*_1_ and *c*_2_ are the cognitive and social learning coefficients, respectively, *r*_1_ and *r*_2_ are uniformly distributed random numbers in the interval [0, 1], **v**_*i, k*_ denotes the velocity vector of particle *i* at iteration *k*, **p**_*i, k*_ denotes the personal-best position of particle *i* at iteration *k*, and **g**_*k*_ denotes the global-best position of the swarm at iteration *k*.

After updating the particle velocity, the particle position is modified according to


$$\begin{array}{l} {\text{x}}_{i,k+1}={\text{x}}_{i,k}+{\text{v}}_{i,k+1}, \end{array}$$ (6)

where **x**_*i, k*_ and **v**_*i, k*+1_ denote the position vector and updated velocity vector of particle *i* at iteration *k*, respectively.

Although conventional PSO provides efficient global search capability, its optimization performance strongly depends on the selection of the inertia weight and learning coefficients. These parameters remain constant throughout the optimization process and therefore cannot adapt to the continuously changing search landscape encountered in dynamic traffic signal optimization. As the swarm gradually converges, the particle diversity decreases, increasing the possibility of premature convergence and entrapment in local optima. These drawbacks inspire the development of the proposed Novelty-Aware Multi-Strategy Particle Swarm Optimization algorithm. It adopts the adaptive novelty search, elite-guided learning, and reinforcement learning based adaptive optimization strategy selection to improve the exploration and convergence performance.

### 3.5 Proposed novelty-aware multi-strategy particle swarm optimization

The conventional Particle Swarm Optimization algorithm evaluates candidate solutions solely according to the objective function value. As the optimization progresses, particles gradually converge toward the global best solution, reducing population diversity and increasing the likelihood of premature convergence. This limitation becomes significant in traffic signal optimization because the search space is highly non-linear and contains numerous local optima. To overcome these limitations, this study proposes a PPO-NAMSPSO algorithm that simultaneously considers solution quality and behavioral diversity during the optimization process.

Unlike conventional PSO, every particle is evaluated using both its traffic performance and its behavioral uniqueness. The behavioral characteristics of the *i*^th^ particle at iteration *k* are represented by


$$\begin{array}{l} {\text{B}}_{i,k}=\left[W_{i,k},Q_{i,k}\right], \end{array}$$ (7)

where *W*_*i, k*_ and *Q*_*i, k*_ denote the average waiting time and average queue length produced by the decoded genome of particle *i* at iteration *k* after microscopic traffic simulation.

For every particle, the Euclidean distance between its behavior descriptor and each archived behavior is calculated as


$$\begin{array}{l} d_{i,j,k}=||{\text{B}}_{i,k}-{\text{B}}_{j}^{\text{arch}}||, \end{array}$$ (8)

where **B**_*i, k*_ denotes the behavior descriptor of particle *i* at iteration *k*, and ${\text{B}}_{j}^{\text{arch}}$ denotes the *j*^th^ behavior descriptor stored in the novelty archive.


$$\begin{array}{l} N_{i,k}=\frac{1}{\left|A_{k}\right|}\underset{{\text{B}}_{j}^{\text{arch}}\in A_{k}}{\sum }d_{i,j,k}, \end{array}$$ (9)

where $A_{k}$ denotes the behavior archive at iteration *k* and $\left|A_{k}\right|$ denotes its current number of stored behavioral descriptors. During the initial iterations, the novelty score is computed using the current archive size rather than the maximum archive capacity *m* = 100. If the archive is empty, the novelty score is assigned its maximum normalized value to encourage exploration.

Instead of selecting particles solely according to waiting time, the proposed algorithm evaluates every candidate solution using a composite fitness function that simultaneously considers traffic delay, queue formation, and behavioral novelty. The fitness of the *i*^th^ particle is calculated as


$$\begin{array}{l} F_{i,k}=W_{i,k}+q_{w}Q_{i,k}-n_{w}N_{i,k}, \end{array}$$ (10)

where *F*_*i, k*_ denotes the composite fitness of particle *i* at iteration *k*, while *q*_*w*_ and *n*_*w*_ denote the queue weighting coefficient and novelty weighting coefficient, respectively.

These coefficients remain fixed throughout the optimization process, while the PPO agent dynamically selects the optimization strategy at each iteration. The composite fitness function is used only for particle ranking and updating the personal best and global best, whereas the final optimization objective remains the minimization of the average vehicle waiting time.

Instead of guiding the entire swarm using only the global best particle, the proposed algorithm constructs an elite solution pool. The elite pool is defined as


$$\begin{array}{l} E_{k}=\left\{{\text{p}}_{e_{1},k},{\text{p}}_{e_{2},k},\ldots ,{\text{p}}_{e_{N_{E}},k}\right\}, \end{array}$$ (11)

where *N*_*E*_ denotes the elite pool size. The elite pool is constructed from the *N*_*E*_ highest-ranked personal-best solutions at iteration *k*. Its size is determined during the offline Optuna–TPE hyperparameter optimization and remains fixed throughout each optimization run.

A stagnation condition is detected when the global-best waiting time does not improve for four consecutive optimization iterations (*patience* = 4). When stagnation is detected, the recovery mechanism is activated. The number of particles reinitialized during stagnation recovery is defined as


$$\begin{array}{l} N_{\text{restart}}=\left\lfloor 0.5n\right\rfloor , \end{array}$$ (12)

where *n* denotes the swarm population size. The *N*_restart_ worst-performing particles are then reinitialized. The floor operation ensures an integer number of particles; therefore, for the tuned population size *n* = 17, *N*_restart_ = 8 particles are reinitialized. The selected particles are assigned randomly generated feasible solutions while their corresponding velocities are reset to zero according to


$$\begin{array}{l} {\text{x}}_{i,k+1}=\text{L}+{\text{r}}_{i,k}⊙\left(\text{U}-\text{L}\right), \end{array}$$ (13)

where **r**_*i, k*_ is a uniformly distributed random vector whose elements lie in the interval [0, 1], **L** and **U** denote the lower and upper bounds of the optimization variables, respectively, and ⊙ denotes element-wise multiplication.

By integrating behavioral novelty, adaptive fitness evaluation, elite-guided particle learning, stagnation recovery, and multiple search strategies within a unified optimization framework, the proposed PPO-NAMSPSO algorithm effectively balances exploration and exploitation while maintaining swarm diversity and improving convergence robustness.

#### 3.5.1 Novelty-guided exploration operator

To enhance exploration, the novelty-guided operator perturbs a subset of particles toward previously unexplored regions of the search space by moving them along randomly generated unit directions. The particle position is updated as


$$\begin{array}{l} {\text{x}}_{i,k+1}={\text{x}}_{i,k}+\alpha {\text{u}}_{i,k}, \end{array}$$ (14)

where **x**_*i, k*_ denotes the current position of particle *i* at iteration *k*, **u**_*i, k*_ denotes a randomly generated unit direction vector, and α is the exploration step size. In this study, α = 0.15. After the update, the particle velocity is reset to zero, and the particle position is clipped to remain within the feasible search space.

#### 3.5.2 Elite-guided search operator

The elite-guided operator promotes exploitation by directing particles toward high-quality solutions maintained in the elite pool. When the PPO agent selects the elite-guided action (*a*_*k*_ = 2), the particle velocity is updated according to


$$\begin{array}{l} {\text{v}}_{i,k+1}=0.3{\text{v}}_{i,k}+2.2r_{i,k}\left({\text{e}}_{i,k}-{\text{x}}_{i,k}\right), \end{array}$$ (15)

where **v**_*i, k*_ denotes the current velocity vector of particle *i* at iteration *k*, *r*_*i, k*_ is a uniformly distributed random number in the interval [0, 1], and **e**_*i, k*_ denotes the elite solution selected for particle *i* from the elite pool at iteration *k*. The updated velocity is subsequently used to obtain the corresponding particle position according to the position-update rule in Equation 6.

#### 3.5.3 Mutation-based search operator

To improve population diversity and prevent premature convergence, the mutation operator introduces stochastic perturbations into the particle trajectory. The velocity update is first computed as


$$\begin{array}{l} {\text{v}}_{i,k+1}=0.6{\text{v}}_{i,k}+1.8r_{i,k}\left({\text{g}}_{k}-{\text{x}}_{i,k}\right), \end{array}$$ (16)

The particle position is then updated according to


$$\begin{array}{l} {\text{x}}_{i,k+1}={\text{x}}_{i,k}+{\text{v}}_{i,k+1}+{\text{M}}_{k}, \end{array}$$ (17)

where **g**_*k*_ denotes the global-best position of the swarm at iteration *k*, *r*_*i, k*_ is a uniformly distributed random number in the interval [0, 1], and **M**_*k*_ represents the Gaussian mutation vector applied at iteration *k*.

The mutation vector is sampled from a zero-mean Gaussian distribution with a standard deviation of 0.15, i.e.,


$$\begin{array}{l} {\text{M}}_{k}\sim N\left(0,0.15^{2}\text{I}\right), \end{array}$$ (18)

where **I** denotes the identity matrix.

The coefficients used in the elite-guided and mutation-based update operators were determined empirically through preliminary experiments to achieve a balanced exploration–exploitation trade-off. These coefficients remained fixed throughout all experiments and were not included in the Optuna hyperparameter optimization process.

#### 3.5.4 Local search operator

To refine promising solutions during the exploitation phase, a local search operator applies a small Gaussian perturbation around the current particle position. The update is defined as


$$\begin{array}{l} {\text{x}}_{i,k+1}={\text{x}}_{i,k}+\delta_{k}, \end{array}$$ (19)

where ***δ***_*k*_ denotes a Gaussian perturbation vector with a small variance, enabling fine-grained local exploration around the current solution. The updated particle position is subsequently restricted to the feasible search region. The perturbation vector is sampled from a zero-mean Gaussian distribution with a standard deviation of 0.01, i.e.,


$$\begin{array}{l} \delta_{k}\sim N\left(0,0.01^{2}\text{I}\right), \end{array}$$ (20)

where **I** denotes the identity matrix.

### 3.6 PPO-based adaptive strategy selection

#### 3.6.1 Optimization state

Although the proposed PPO-NAMSPSO algorithm significantly enhances the search capability through novelty-guided exploration, elite-guided learning, and stagnation recovery, selecting an appropriate optimization strategy at different stages of the search process remains a challenging task. Figure 3 illustrates the PPO state–action–reward architecture adopted in the proposed framework. Applying a single search strategy throughout the optimization process may reduce exploration capability during the early stages of the search or slow convergence during the later stages of optimization. To overcome this limitation, the proposed framework incorporates Proximal Policy Optimization (PPO) to adaptively select the most suitable optimization strategy according to the current optimization state. The PPO agent continuously interacts with the optimization environment and learns a policy that maximizes long-term optimization performance by selecting the most effective search operator at each optimization iteration.

**Figure 3:**
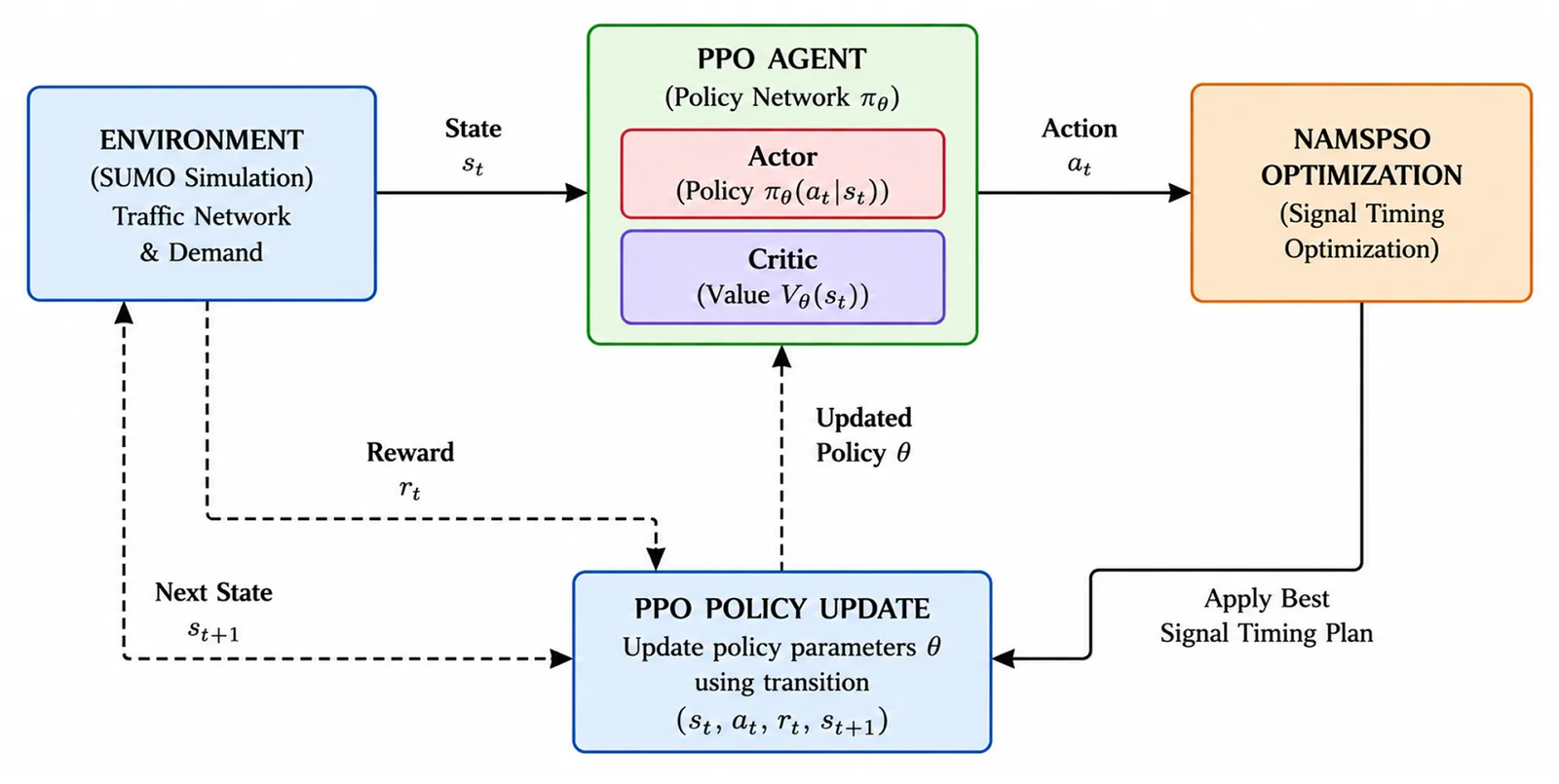
PPO state–action–reward architecture for adaptive optimization strategy selection.

The optimization state observed by the PPO agent at iteration *k* is represented as


$$\begin{array}{l} {\text{S}}_{k}=\left[s_{1,k},s_{2,k},\ldots ,s_{8,k}\right], \end{array}$$ (21)

$$\begin{array}{l} {\text{Div}}_{k}=\frac{1}{D}\overset{D}{\underset{d=1}{\sum }}\mathrm{Std}\left(x_{1d,k},x_{2d,k},\ldots ,x_{nd,k}\right), \end{array}$$ (22)

$$\begin{array}{l} s_{2,k}=\mathrm{clip}\left({\text{Div}}_{k},0,1\right), \end{array}$$ (23)

where the state vector consists of eight normalized optimization features describing the current search behavior. Specifically, *s*_1_ represents the optimization progress, while *s*_2_ represents the normalized swarm diversity, computed as the average standard deviation of particle positions across all decision variables and clipped to the interval [0, 1]. Higher values indicate greater exploration, whereas lower values indicate swarm convergence. Furthermore, *s*_3_ represents the normalized best waiting time, *s*_4_ denotes the normalized average queue length, *s*_5_ represents the average novelty score, *s*_6_ denotes the normalized stagnation level measured using consecutive iterations without improvement, *s*_7_ represents the normalized improvement in the global best waiting time, and *s*_8_ denotes the normalized operator selection frequency, computed as the ratio of the number of times the currently selected optimization strategy appears in the recent operator history to the total number of recorded operator selections. The optimization state variables are summarized in Table 2.

**Table 2:** PPO optimization state variables.

State	Code	Description
*s*_1_	progress	Optimization progress
*s*_2_	diversity	Swarm diversity
*s*_3_	best_wait / 80	Normalized best waiting time
*s*_4_	avg_queue / 25	Normalized average queue length
*s*_5_	avg_novelty	Average novelty score
*s*_6_	no_improve / patience	Normalized stagnation level
*s*_7_	last_improvement / 10	Normalized improvement in the global best waiting time
*s*_8_	operator_success	Normalized operator selection frequency

#### 3.6.2 Action space

The PPO policy network predicts a discrete optimization action represented as


$$\begin{array}{l} a_{k}\in \left\{0,1,2,3,4,5\right\}, \end{array}$$ (24)

where *a*_*k*_ denotes the search strategy selected by the PPO agent at optimization iteration *k*. The standard PSO update is applied at every optimization iteration as the base population update, independently of the selected action. The selected action determines whether an additional search mechanism is applied after the standard PSO update. Specifically,

- *a*_*k*_ = 0: No additional search operator; only the standard PSO update is applied.
- *a*_*k*_ = 1: Novelty-guided exploration applied after the standard PSO update.
- *a*_*k*_ = 2: Elite-guided PSO mode, in which the elite pool is used for leader selection within the PSO update.
- *a*_*k*_ = 3: Mutation-based search applied after the standard PSO update.
- *a*_*k*_ = 4: Restart strategy applied after the standard PSO update to recover from optimization stagnation.
- *a*_*k*_ = 5: Local search applied after the standard PSO update for solution refinement.

At each optimization iteration, the standard PSO velocity and position updates defined by Equations 5, 6 are first applied to the swarm population. The PPO action then determines the additional search mechanism. When *a*_*k*_ = 0, no additional operator is applied. For *a*_*k*_∈{1, 3, 4, 5}, the corresponding novelty-guided, mutation-based, restart, or local-search operator is applied to the population after the standard PSO update. For *a*_*k*_ = 2, the elite pool is incorporated into the PSO leader-selection mechanism rather than applying a separate post-PSO update.

Thus, the PPO agent does not replace the standard PSO update. Instead, it adaptively determines the additional search behavior while the standard PSO mechanism provides the base population movement. This design enables the optimization process to balance exploration and exploitation according to the current optimization state.

#### 3.6.3 Reward function

The PPO agent subsequently receives a reinforcement learning reward that reflects the optimization performance achieved after applying the selected search strategy. The reward function simultaneously encourages reduction in traffic delay, preservation of swarm diversity, behavioral exploration, and avoidance of optimization stagnation. The reward is formulated as


$$\begin{array}{l} R_{k}=5\Delta W_{k}-0.2Q_{k}+0.1{\text{Div}}_{k}+0.05{\bar{N}}_{k}-0.2S_{g,k}, \end{array}$$ (25)

where Δ*W*_*k*_ denotes the improvement in the global-best waiting time at iteration *k*, *Q*_*k*_ denotes the normalized average queue length of the swarm at iteration *k*, Div_*k*_ represents the normalized swarm diversity at iteration *k*, ${\bar{N}}_{k}$ denotes the average novelty score at iteration *k*, and *S*_*g, k*_ represents the normalized stagnation level at iteration *k*. The first term rewards reductions in the global-best waiting time, whereas the remaining terms encourage balanced exploration while penalizing excessive queueing and prolonged stagnation. To maintain stable reinforcement learning, the reward is clipped to the interval [−5, 5] after each optimization iteration before being used for PPO learning.

The reward coefficients were selected through manual empirical calibration based on the relative importance and numerical scale of the constituent signals. A higher coefficient of 5 was assigned to the improvement in the best waiting time because it represents the primary optimization objective, whereas the queue, diversity, novelty, and stagnation terms were assigned smaller coefficients (0.20, 0.10, 0.05, and 0.20, respectively) to provide supporting behavioral signals without dominating the primary improvement term. These coefficients were kept fixed across all experiments and were not retuned for individual traffic scenarios or random seeds.

All reward components were normalized before aggregation so that the coefficient magnitudes represent their intended relative importance rather than differences in numerical scale. The coefficients were kept fixed during PPO training and were not modified during the final evaluation experiments. Finally, the resulting reward was clipped to [−5, 5] after each optimization iteration to limit excessively large reward values and improve the numerical stability of PPO training.

#### 3.6.4 Policy learning

During training, the PPO agent continuously observes the optimization state, selects an optimization strategy, receives the corresponding reward from the optimization environment, and updates its policy to maximize the cumulative reward over successive optimization iterations.

Formally, let


$$\rho_{k}\left(\theta \right)=\frac{\pi_{\theta }\left(a_{k}|{\text{s}}_{k}\right)}{\pi_{\theta_{\text{old}}}\left(a_{k}|{\text{s}}_{k}\right)}$$

denote the probability ratio between the current and previous policy, and let Â_*k*_ denote the advantage estimate obtained via Generalized Advantage Estimation (GAE) with γ = 0.99 and λ = 0.95. The clipped surrogate objective is


$$L^{\text{CLIP}}(\theta )=E_{k}\text{​}\left[\min\text{​}(\rho_{k}(\theta ){\hat{A}}_{k},\;\mathrm{clip}(\rho_{k}(\theta ),1-\epsilon ,1+\epsilon ){\hat{A}}_{k})\right],$$ (26)

where ϵ = 0.20 is the PPO clipping range. The full training objective additionally includes a value-function loss and entropy bonus:


$$L(\theta )=E_{k}\text{​}\left[L^{\text{CLIP}}(\theta )-c_{V}{\left(V_{\theta }(s_{k})-V_{k}^{\text{target}}\right)}^{2}+c_{H}H(\pi_{\theta }(\cdot |s_{k}))\right],$$ (27)

where *V*_θ_(**s**_*k*_) denotes the critic's value estimate, $V_{k}^{\text{target}}$ denotes the target value at iteration *k*, *H*(·) is the policy entropy, *c*_*V*_ is the value-function loss coefficient, and *c*_*H*_ = 0.005 is the entropy coefficient.

The policy and value network parameters are updated by gradient ascent using the Adam optimizer with learning rate $\alpha_{\theta }=3\times 10^{-4}$:


$$\begin{array}{l} \theta \leftarrow \theta +\alpha_{\theta }\nabla_{\theta }L\left(\theta \right), \end{array}$$ (28)

where θ denotes the trainable parameters of the PPO policy and value networks, and $\alpha_{\theta }=3\times 10^{-4}$ is the Adam learning rate.

Training proceeds for 15,000 interaction timesteps, accumulated over repeated episodes of *K* = 8 PPO-NAMSPSO optimization iterations, using the reward *R*_*k*_ defined in Equation 25. Once training terminates, the resulting parameters are fixed as θ^*^, and $\pi_{\theta^{*}}$ is subsequently used only for inference during the deployment procedure described in Algorithm 1.

Algorithm 1 **Offline PPO training for adaptive operator selection.**

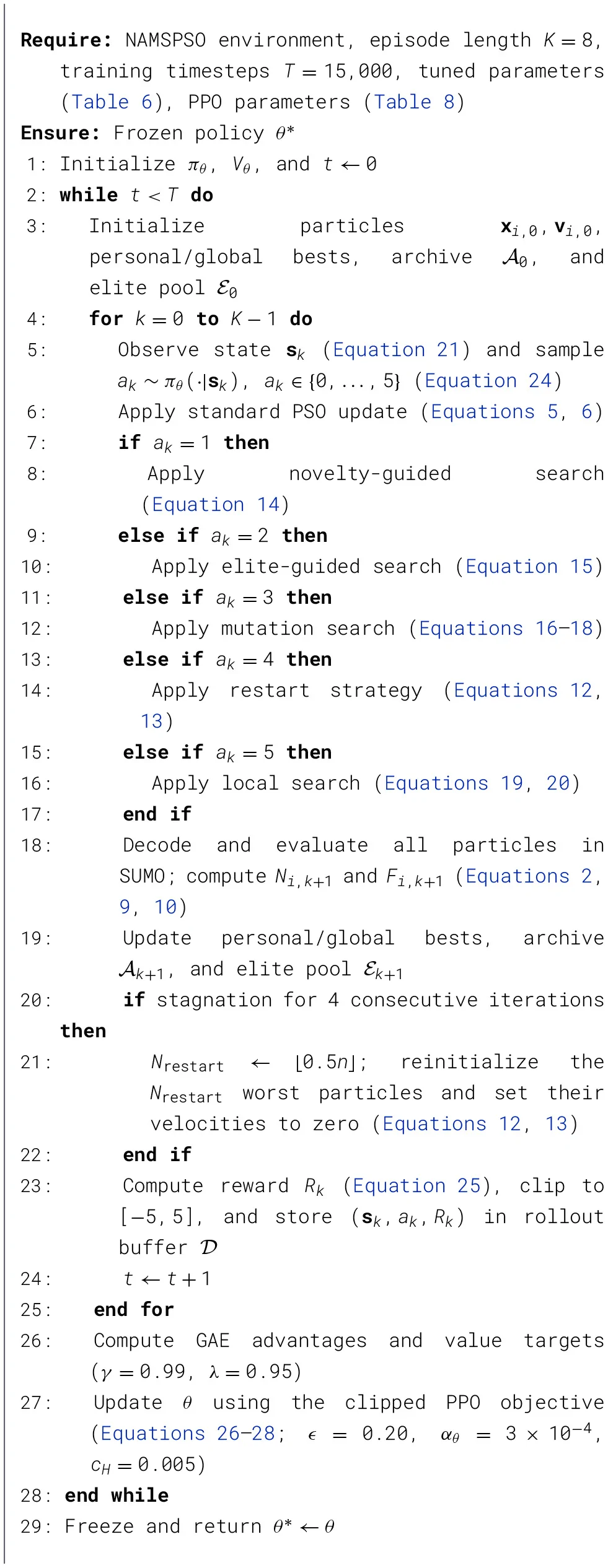

### 3.7 Hyperparameter initialization using Optuna–TPE

The performance of swarm intelligence algorithms is highly dependent on the selection of optimization hyperparameters. Conventional approaches generally determine these parameters through manual trial-and-error tuning, which often produces inconsistent optimization performance under different traffic conditions and significantly increases computational effort. To overcome this limitation, the proposed framework employs the Optuna optimization framework with the Tree-structured Parzen Estimator (TPE) sampler to automatically determine an effective baseline hyperparameter configuration before reinforcement learning is applied. The optimized hyperparameters are subsequently used to initialize the proposed PPO-NAMSPSO algorithm.

In this study, the Optuna framework utilizes the Tree-structured Parzen Estimator (TPE) sampler to optimize the baseline hyperparameters by minimizing the average vehicle waiting time obtained from the SUMO microscopic traffic simulator. Each trial represents a unique hyperparameter configuration, and the corresponding traffic signal timing plans are evaluated through microscopic traffic simulation. The hyperparameter configuration that produces the minimum optimization objective is selected as the baseline configuration for the proposed optimization framework.

The optimization hyperparameter vector is represented as


$$\begin{array}{l} \Theta =\left[n,\omega ,c_{1},c_{2},q_{w},n_{w},{\text{noise}}_{\text{scale}},v_{\max},N_{E}\right], \end{array}$$ (29)

where *n* denotes the swarm population size, ω denotes the inertia weight, *c*_1_ and *c*_2_ denote the cognitive and social learning coefficients, *q*_*w*_ and *n*_*w*_ denote the queue weighting coefficient and novelty weighting coefficient, respectively, noise_scale_ denotes the stochastic perturbation magnitude applied during particle updating, *v*_max_ denotes the maximum particle velocity, and *N*_*E*_ denotes the elite pool size.

The hyperparameter optimization objective is formulated as


$$\begin{array}{l} \Theta^{*}=\arg\underset{\Theta }{\min}J\left(\Theta \right), \end{array}$$ (30)

where *J*(***Θ***) denotes the traffic optimization objective obtained after evaluating the corresponding hyperparameter configuration using the SUMO microscopic traffic simulator. In this study, the optimization objective is defined as minimizing the average vehicle waiting time.

The Tree-structured Parzen Estimator (TPE) sampler selects the next hyperparameter configuration according to


$$\begin{array}{l} \Theta_{\text{next}}=\arg\underset{\Theta }{\max}\frac{\ell_{\text{good}}\left(\Theta \right)}{\ell_{\text{bad}}\left(\Theta \right)}. \end{array}$$ (31)

where ℓ_good_(***Θ***) and ℓ_bad_(***Θ***) denote the likelihood functions modeling promising and non-promising hyperparameter configurations, respectively. This acquisition strategy favors hyperparameter configurations with a higher probability of producing improved optimization performance while reducing the number of required optimization trials.

After completing all optimization trials, the best-performing hyperparameter configuration is represented as


$$\begin{array}{l} \Theta^{*}=\arg\underset{\Theta \in T}{\min}J\left(\Theta \right), \end{array}$$ (32)

where T denotes the set of hyperparameter configurations evaluated during the Optuna–TPE search and *J*(***Θ***) denotes the corresponding average vehicle waiting time.

where each parameter corresponds to the optimal baseline hyperparameter configuration identified by the Optuna–TPE optimization process. The optimized hyperparameters include the swarm population size (*n*), inertia weight (ω), cognitive coefficient (*c*_1_), social coefficient (*c*_2_), queue weighting coefficient (*q*_*w*_), novelty weighting coefficient (*n*_*w*_), noise scale (noise_scale_), maximum particle velocity (*v*_max_), and elite pool size (*N*_*E*_). These hyperparameters were fixed throughout PPO training and all comparative experiments, while the PPO agent learned only the adaptive optimization strategy selection policy.

The optimized hyperparameter vector obtained through Optuna–TPE is subsequently used to initialize the proposed PPO-NAMSPSO optimizer before reinforcement learning begins. During the optimization process, these baseline hyperparameters remain fixed, whereas the PPO agent dynamically observes the optimization state and selects the most appropriate optimization strategy from the available search operators. Consequently, Optuna–TPE determines the baseline hyperparameters before optimization, whereas PPO dynamically selects optimization strategies during the search process.

### 3.8 Hybrid PPO-NAMSPSO optimization algorithm

The proposed optimization framework integrates Proximal Policy Optimization (PPO) with the Novelty-Aware Multi-Strategy Particle Swarm Optimization (NAMSPSO) algorithm to achieve adaptive traffic signal optimization. Figure 4 illustrates the internal workflow of the proposed PPO-NAMSPSO optimization framework. Unlike conventional Particle Swarm Optimization (PSO), which employs a fixed particle update mechanism throughout the optimization process, the proposed framework uses a trained PPO policy to adaptively select an additional search strategy according to the current optimization state. At each optimization iteration, the standard PSO velocity and position update is first performed, after which the PPO-selected strategy is applied as an additional search mechanism. When the PPO selects the baseline action (*a*_*k*_ = 0), no additional search operator is applied. For *a*_*k*_∈{1, …, 5}, the corresponding novelty-guided, elite-guided, mutation-based, restart, or local-search strategy is applied following the standard PSO update. This adaptive integration enables the optimizer to balance global exploration and local exploitation, preserve swarm diversity, and improve convergence stability under dynamic traffic conditions.

**Figure 4:**
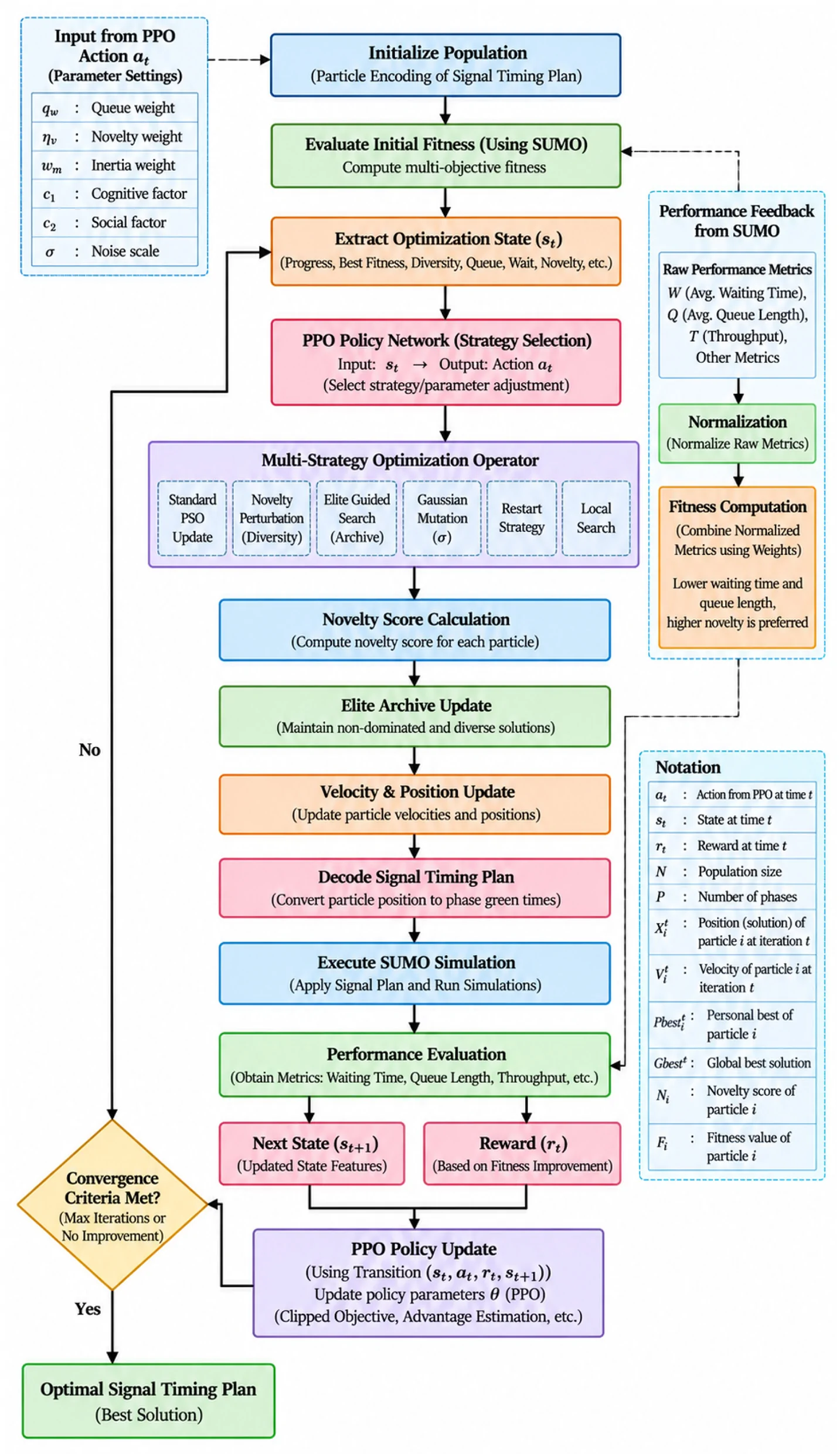
Internal workflow of the proposed PPO-NAMSPSO optimization framework.

Algorithm 2 summarizes the optimization stage of the proposed PPO-NAMSPSO framework. The process begins by initializing the particle population, velocities, personal bests, global best, behavior archive, and elite pool. At each iteration, the current optimization state **s**_*k*_ is provided to the trained PPO policy $\pi_{\theta^{*}}$, which selects an additional search strategy *a*_*k*_. The standard PSO update is then performed for all particles, followed by the selected additional search operator. Each updated particle is evaluated using the SUMO simulator, after which its traffic performance, novelty score, and composite fitness are calculated. The personal best, global best, behavior archive, and elite pool are subsequently updated, and stagnation recovery is activated when necessary. The reinforcement learning reward is then computed based on the improvement in waiting time, queue length, swarm diversity, novelty, and stagnation. The resulting state **s**_*k*+1_ is used in the subsequent iteration.

Algorithm 2 **PPO-NAMSPSO optimization procedure (deployment phase).**

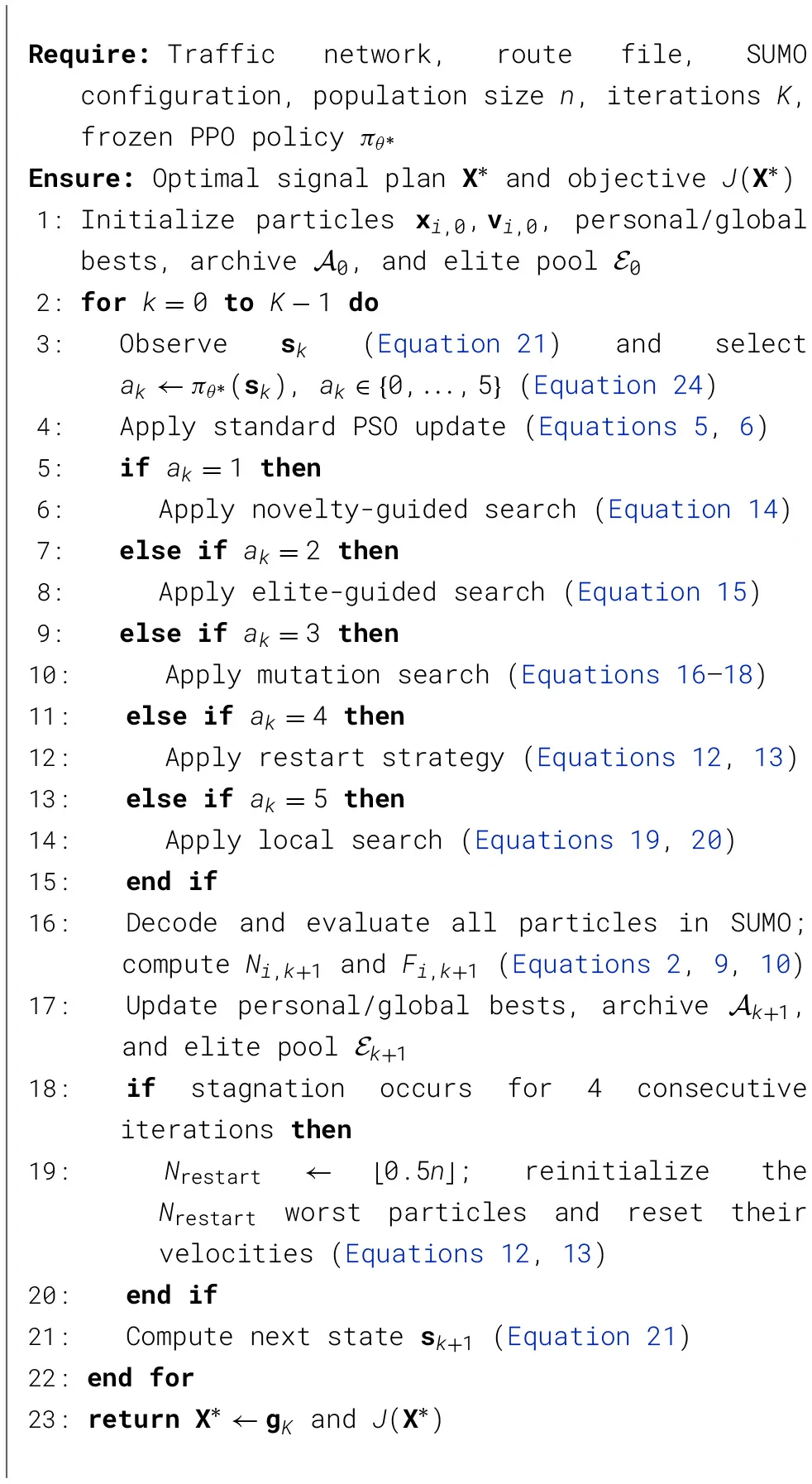

The PPO policy is trained prior to the optimization stage using the reinforcement learning environment and reward formulation described in Section 3.6.4.The resulting policy parameters θ^*^ remain fixed during the PPO-NAMSPSO optimization process; no further PPO policy update is performed during this stage. Thus, PPO adaptively selects the additional search strategy, while the standard PSO update and the selected PPO-NAMSPSO operator perform the population search.

### 3.9 Computational complexity and convergence analysis

The computational efficiency of the proposed PPO-NAMSPSO framework is determined by the combined computational cost of particle initialization, microscopic traffic simulation, behavioral novelty evaluation, elite solution selection, reinforcement learning inference, and multi-strategy particle updating. During each optimization iteration, the PPO agent observes the current optimization state and selects one optimization strategy, which is subsequently executed by the PPO-NAMSPSO algorithm to update the swarm population.

Consequently, the computational complexity of a single optimization iteration is expressed as


$$\begin{array}{l} O\left(nD+nm+n\log n+nT_{\text{sim}}\right), \end{array}$$ (33)

where *n* denotes the swarm population size, *D* is the number of optimization variables, *m* is the novelty archive size, and *T*_sim_ denotes the computational cost of one SUMO microscopic traffic simulation.

Considering the complete optimization process over *K* iterations, the overall computational complexity becomes


$$\begin{array}{l} O\left(K\left(nD+nm+n\log n+nT_{\text{sim}}\right)\right), \end{array}$$ (34)

The proposed PPO-NAMSPSO framework improves convergence by combining novelty-aware exploration, elite-guided learning, adaptive strategy selection, and stagnation recovery. These complementary mechanisms maintain swarm diversity during the early optimization stage while improving solution refinement during later iterations, resulting in stable convergence and reducing the likelihood of premature convergence.

Overall, the proposed PPO-NAMSPSO framework provides efficient global exploration and reliable local exploitation while maintaining convergence stability, making it suitable for adaptive traffic signal optimization. For clarity and to ensure consistency of mathematical notation throughout the proposed framework, Table 3 summarizes the principal equations, symbols, and their corresponding meanings.

**Table 3:** Equation index: symbol, equation number, and description.

Equation number	Symbol/LHS	Meaning
Equation 1	**X** = [*x*_1_, …, *x*_*D*_]	Candidate solution (traffic signal genome)
Equation 2	(CL, GS) = *f*_decode_(**X**)	Genome decoding into cycle length/green split
Equation 3	${\text{X}}^{*}=\arg\min_{\text{X}}J\left(\text{X}\right)$	Overall optimization objective
Equation 4	*J*(**X**) = *W*(**X**)	Objective = average vehicle waiting time
Equation 5	**v**_*i, k*+1_ = ω**v**_*i, k*_+*c*_1_*r*_1_(**p**_*i, k*_−**x**_*i, k*_)+*c*_2_*r*_2_(**g**_*k*_−**x**_*i, k*_)	Standard PSO velocity update
Equation 6	**x**_*i, k*+1_ = **x**_*i, k*_+**v**_*i, k*+1_	Standard PSO position update
Equation 7	**B**_*i, k*_ = (*W*_*i, k*_, *Q*_*i, k*_)	Behavioral descriptor (wait time, queue)
Equation 8	$d_{i,j,k}=∥{\text{B}}_{i,k}-{\text{B}}_{j}^{\text{arch}}∥$	Distance to archived behavior
Equation 9	$N_{i,k}=\frac{1}{\left|A_{k}\right|}\sum d_{i,j,k}$	Novelty score
Equation 10	*F*_*i, k*_ = *W*_*i, k*_+*q*_*w*_*Q*_*i, k*_−*n*_*w*_*N*_*i, k*_	Composite fitness
Equation 11	$E_{k}=\left\{{\text{p}}_{e1,k},\ldots ,{\text{p}}_{eN_{E},k}\right\}$	Elite pool
Equation 12	*N*_restart_ = ⌊0.5*n*⌋	Number of particles reinitialized on stagnation
Equation 13	**x**_*i, k*+1_ = **L**+**r**_*i, k*_⊙(**U**−**L**)	Restart reinitialization rule
Equation 14	**x**_*i, k*+1_ = **x**_*i, k*_+α**u**_*i, k*_	Novelty-guided exploration operator
Equation 15	**v**_*i, k*+1_ = 0.3**v**_*i, k*_+2.2*r*_*i, k*_(**e**_*i, k*_−**x**_*i, k*_)	Elite-guided velocity update
Equation 16	**v**_*i, k*+1_ = 0.6**v**_*i, k*_+1.8*r*_*i, k*_(**g**_*k*_−**x**_*i, k*_)	Mutation-based velocity update
Equation 17	**x**_*i, k*+1_ = **x**_*i, k*_+**v**_*i, k*+1_+**M**_*k*_	Mutation-based position update
Equation 18	${\text{M}}_{k}\sim N\left(0,0.15^{2}\text{I}\right)$	Mutation noise vector
Equation 19	**x**_*i, k*+1_ = **x**_*i, k*_+***δ***_*k*_	Local search position update
Equation 20	$\delta_{k}\sim N\left(0,0.01^{2}\text{I}\right)$	Local search perturbation vector
Equation 21	**s**_*k*_ = (*s*_1, *k*_, …, *s*_8, *k*_)	PPO optimization state vector
Equation 22	${\text{Div}}_{k}=\frac{1}{D}\overset{D}{\underset{d=1}{\sum }}\text{Std}\left(\cdot \right)$	Swarm diversity
Equation 23	*s*_2, *k*_ = clip(Div_*k*_, 0, 1)	Normalized diversity feature
Equation 24	*a*_*k*_∈{0, 1, 2, 3, 4, 5}	PPO discrete action (operator selection)
Equation 25	$R_{k}=5\Delta W_{k}-0.2Q_{k}+0.1{\text{Div}}_{k}+0.05{\bar{N}}_{k}-0.2S_{g,k}$	PPO reward function
Equation 26	*L*^CLIP^(θ)	Clipped PPO surrogate objective
Equation 27	$L\left(\theta \right)=E\left[L^{\text{CLIP}}\left(\theta \right)-c_{V}{\left(\cdot \right)}^{2}+c_{H}H\left(\cdot \right)\right]$	Full PPO training loss (value + entropy)
Equation 28	θ←θ+α_θ_∇_θ_*L*(θ)	PPO gradient-ascent update
Equation 29	Θ = [*n*, ω, *c*_1_, *c*_2_, *q*_*w*_, *n*_*w*_, noise_scale_, *v*_max_, *N*_*E*_]	Optuna hyperparameter vector
Equation 30	$\Theta^{*}=\arg\min_{\Theta }J\left(\Theta \right)$	Hyperparameter optimization objective
Equation 31	$\Theta_{\text{next}}=\arg\max_{\Theta }\ell_{\text{good}}\left(\Theta \right)/\ell_{\text{bad}}\left(\Theta \right)$	TPE acquisition rule
Equation 32	$\Theta^{*}=\arg\min_{\Theta \in C}J\left(\Theta \right)$	Best hyperparameter config over trial set T
Equation 33	*O*(*nD*+*nm*+*n*log*n*+*nT*_sim_)	Per-iteration computational complexity
Equation 34	*O*(*K*(*nD*+*nm*+*n*log*n*+*nT*_sim_))	Full-run computational complexity

## 4 Experimental setup

### 4.1 Simulation environment

The proposed PPO-NAMSPSO framework was implemented using the Simulation of Urban Mobility (SUMO) microscopic traffic simulator, which provides a realistic environment for modeling urban traffic dynamics and evaluating adaptive traffic signal control strategies. Real-time communication between the traffic simulator and the optimization framework was established through the Traffic Control Interface (TraCI), allowing the optimizer to continuously collect traffic information and update traffic signal timings during simulation.

All experiments were conducted on a workstation equipped with an Intel Core i7-12700H CPU, 16 GB RAM, and the Windows 11 operating system. The optimization framework was implemented using Python 3.11 (Python Software Foundation, Wilmington, DE, USA), SUMO 1.20.0 [German Aerospace Center (DLR)/Eclipse SUMO, Berlin, Germany], TraCI [German Aerospace Center (DLR), as part of SUMO, Berlin, Germany], Stable-Baselines3 2.3.0 [DLR-RM, German Aerospace Center (DLR), Oberpfaffenhofen-Weßling Germany], Optuna 4.0.0 (Preferred Networks, Inc., Tokyo, Japan), and Gymnasium 0.29 (Farama Foundation, USA). Table 4 summarizes the simulation environment and software configuration used throughout the experssimental evaluation.

**Table 4:** SUMO simulation configuration used for experimental evaluation.

Parameter	Value
Traffic simulator	SUMO 1.20.0
Programming language	Python 3.11
RL library	Stable-Baselines3 2.3.0
Hyperparameter optimization	Optuna 4.0.0 (TPE)
Environment interface	TraCI
Simulation duration	3600 s
Traffic scenarios	Medium (1350 vehicles), High (2472 vehicles)
Vehicle types	Car (45%), Bike (30%), Bus (15%), Truck (10%)
Route generation	Custom Python script using sumolib shortest-path routing
Route files	Generated once and reused for all experiments
Vehicle insertion	departLane = “best”, departSpeed = “max”

### 4.2 Traffic network configuration

The proposed PPO-NAMSPSO framework was evaluated on two different traffic networks to assess its performance under both controlled benchmark scenarios and realistic urban traffic conditions. A synthetic benchmark network was employed to evaluate the optimization capability of the proposed framework in a structured and repeatable environment, whereas a real-world road network extracted from OpenStreetMap (OSM) was used to investigate its scalability and applicability under practical urban traffic conditions.

#### 4.2.1 Synthetic benchmark network

The synthetic benchmark network consists of a 3 × 3 urban grid comprising nine intersections (J1–J9), as illustrated in Figure 5. The intersections are connected through bidirectional road segments that support traffic movement in both the horizontal and vertical directions. Vehicles enter and exit the network through the boundary intersections, while the interior signalized intersections are controlled by traffic signal controllers whose green phase durations are optimized by the evaluated traffic signal optimization algorithms. The regular network topology provides a controlled, reproducible, and scalable experimental environment for evaluating adaptive traffic signal optimization methods under different traffic demand scenarios.

**Figure 5:**
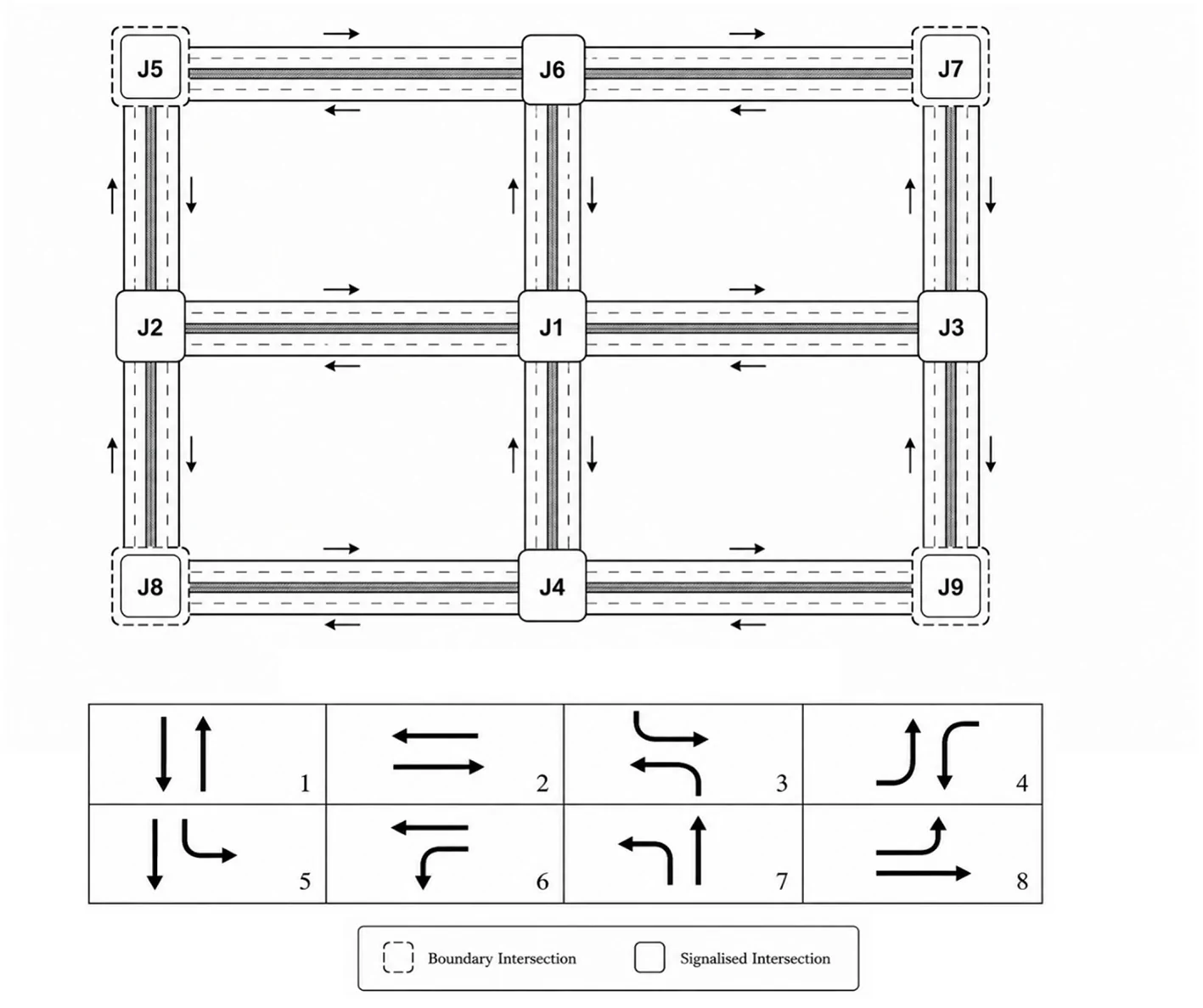
Synthetic 3 × 3 benchmark traffic network used for experimental evaluation.

#### 4.2.2 Real-world OSM network

The proposed PPO-NAMSPSO framework was further evaluated on a real-world road network extracted from OpenStreetMap (OSM), as illustrated in Figure 6, to assess its performance under realistic urban traffic conditions. The OSM data were converted into a SUMO-compatible traffic network using the netconvert utility, preserving the original road geometry, lane connectivity, junction topology, and traffic control infrastructure.

**Figure 6:**
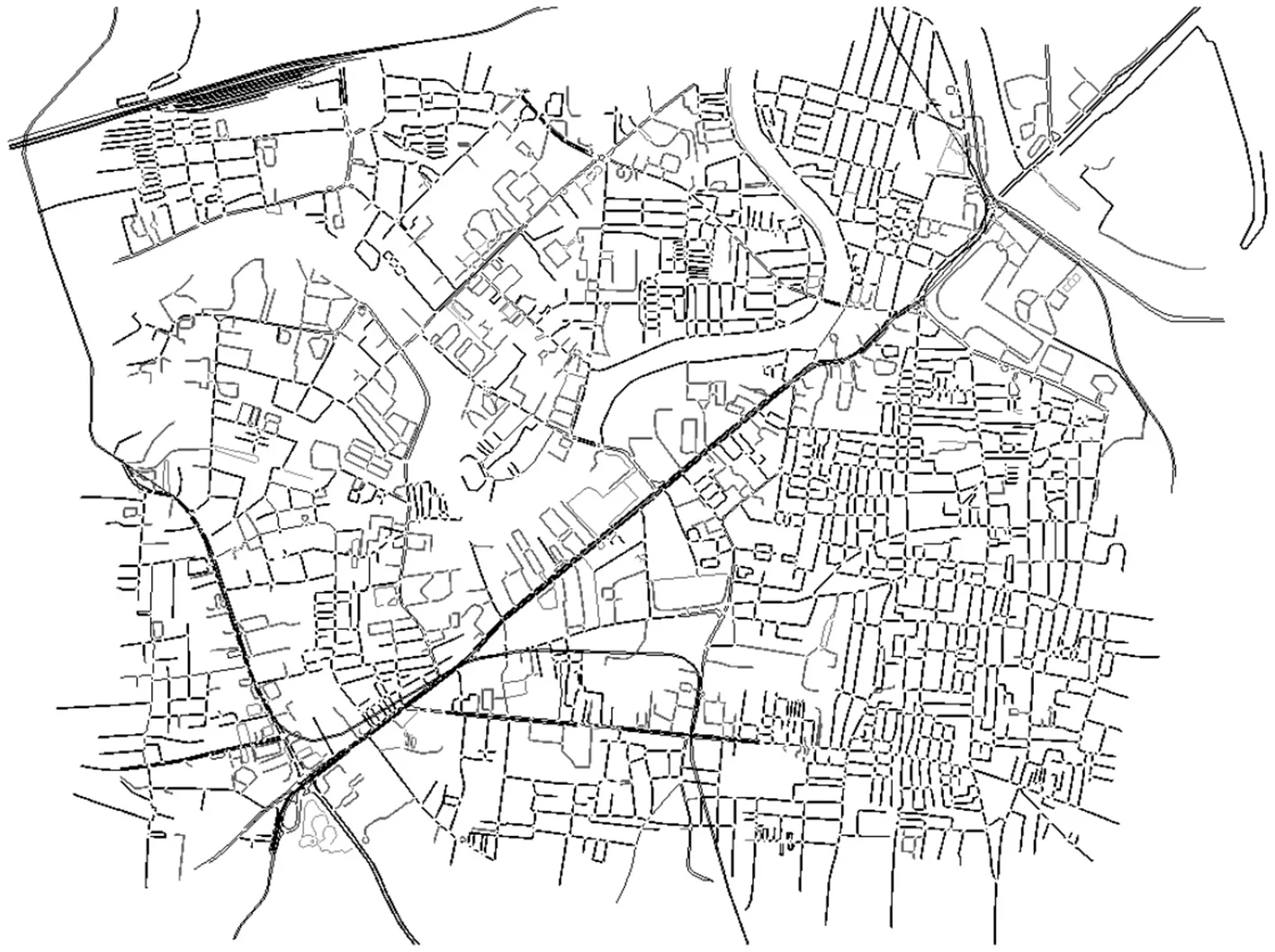
Real-world OpenStreetMap (OSM) road network used for experimental evaluation.

The resulting network represents a heterogeneous urban road system composed of primary, secondary, tertiary, residential, and service roads, thereby capturing the complexity of real-world traffic environments. In contrast to the regular topology of the synthetic benchmark network, the OSM network contains irregular road layouts, varying intersection geometries, heterogeneous lane configurations, and diverse traffic flow patterns. The network includes 25 signalized intersections, all of which were considered during the optimization process to evaluate the scalability and robustness of the proposed PPO-NAMSPSO framework under large-scale urban traffic conditions.

### 4.3 Traffic demand and signal configuration

The proposed PPO-NAMSPSO framework was evaluated under medium and high traffic demand scenarios to assess its performance across different congestion levels. Traffic demand was generated once using a custom Python script developed with the SUMO sumolib library. The script automatically generated feasible origin–destination routes using shortest-path routing and assigned heterogeneous vehicle types consisting of cars (45%), motorcycles (30%), buses (15%), and trucks (10%). Vehicle departures followed a dual-peak traffic distribution over a simulation period of 3600 s. The generated traffic demand consisted of 1,350 vehicles for the medium traffic scenario and 2,472 vehicles for the high traffic scenario. After generation, the resulting route files were kept unchanged and reused for all optimization algorithms and experimental runs to ensure identical traffic demand and a fair comparison. Vehicles were inserted using departLane = “best” and departSpeed = “max”.

Five signalized intersections (J1, J2, J3, J4, and J6) in the synthetic benchmark network were selected for adaptive signal timing optimization. Each intersection was controlled using a fixed four-phase traffic signal cycle comprising two green phases for non-conflicting traffic movements and two yellow transition phases. The initial duration of each green phase was set to 42 s, whereas the yellow transition interval was fixed at 3 s. During optimization, only the durations of the green phases were modified, while the signal phase sequence and yellow transition intervals remained unchanged throughout all experiments.

For the real-world OSM network, the traffic signal controllers generated during the SUMO network conversion process were preserved. The proposed PPO-NAMSPSO framework optimized only the green phase durations while maintaining the original signal sequence and traffic control logic, thereby enabling performance evaluation under realistic urban traffic conditions without modifying the existing intersection control structure. Table 5 summarizes the traffic signal configuration adopted for the synthetic benchmark network.

**Table 5:** Traffic signal phase configuration of the synthetic benchmark network.

Phase	Initial duration (s)	Signal state (J1, J3, J6)	Signal state (J2, J4)	Description
Phase 1	42	GGggrrrrGGG	rrrGGgGGgGg	Green phase for one set of non-conflicting traffic movements.
Phase 2	3	yyyyrrrrGyy	rrrGygyyyyy	Yellow transition following Phase 1.
Phase 3	42	rrrrGGgGGrr	GGGGrGrrrrr	Green phase for the opposing non-conflicting traffic movements.
Phase 4	3	rrrryyyyGrr	yyyGryrrrrr	Yellow transition following Phase 3.

*G* denotes a protected green signal, *g* denotes a permissive green signal, *y* denotes a yellow transition signal, and *r* denotes a red signal. During optimization, only the durations of the green phases were optimized, while the signal phase sequence and yellow transition intervals were kept fixed across all experiments.

### 4.4 Hyperparameter optimization

The Optuna–TPE hyperparameter optimization procedure described in Section 3.6 was executed before PPO training to determine the baseline hyperparameter configuration. Hyperparameter optimization was performed using the Optuna framework with the Tree-Structured Parzen Estimator (TPE) sampler. For computational efficiency, the tuning procedure was conducted using the low-traffic scenario as the calibration scenario. For each optimization algorithm, 20 optimization trials were conducted, with each trial consisting of eight optimization iterations under identical simulation settings. The optimization objective was to minimize the average vehicle waiting time obtained from the SUMO microscopic traffic simulator.

The low-traffic scenario was used only for hyperparameter calibration and was not used as a substitute for the final comparative evaluation scenarios. The best hyperparameter configuration identified during tuning was subsequently fixed and used in the experimental evaluation. Therefore, the objective values reported for the Optuna–TPE tuning stage represent performance under the low-traffic calibration condition and are not directly comparable with the waiting-time values reported in the final benchmark and OSM evaluations.

To reduce computational effort, the MedianPruner strategy was employed with five startup trials, one warmup step, and a pruning interval of one step for early termination of unpromising trials. The optimization study was stored in an SQLite database, allowing interrupted tuning sessions to be resumed without repeating completed trials.

Table 6 summarizes the optimal hyperparameter configuration obtained for the proposed PPO-NAMSPSO framework.

**Table 6:** Optuna–TPE optimized hyperparameters and corresponding best waiting-time objective values obtained under the low-traffic calibration scenario.

Hyperparameter	Optimized value
Particle population (*n*)	17
Inertia weight (ω)	0.7944
Cognitive coefficient (*c*_1_)	1.0128
Social coefficient (*c*_2_)	1.5775
Queue weight (*q*_*w*_)	0.3042
Novelty weight (*n*_*w*_)	0.0986
Noise scale	0.0052
Maximum velocity (*v*_max_)	0.2976
Elite pool size (*N*_*E*_)	3

The best-performing hyperparameter configuration identified using the Optuna–TPE framework consisted of 17 particles, an inertia weight of 0.7944, a cognitive coefficient of 1.0128, a social coefficient of 1.5775, a queue weighting coefficient of 0.3042, a novelty weighting coefficient of 0.0986, a noise scale of 0.0052, a maximum particle velocity of 0.2976, and an elite pool size of 3. This configuration achieved the lowest average vehicle waiting time of 15.55 s and was therefore adopted throughout PPO training and comparative evaluation.

To ensure a fair comparison, all benchmark optimization algorithms were independently tuned using the same Optuna–TPE optimization budget. The resulting optimized configurations are summarized in Table 7.

**Table 7:** Optuna–TPE-optimized hyperparameters of the baseline optimization algorithms.

Algorithm	Best objective value	Best hyperparameters	Trials
PSO	22.5450	Particles = 17, ω = 0.83698, *c*_1_ = 1.31539, *c*_2_ = 2.45064, Noise scale = 0.04767, *v*_max_ = 0.35034	20
PPO-NAMSPSO	15.5524	Particles = 17, ω = 0.79440, *c*_1_ = 1.01284, *c*_2_ = 1.57753, Queue weight = 0.30419, Novelty weight = 0.09865, Noise scale = 0.00523, *v*_max_ = 0.29762, Elite pool size = 3	20
GA	33.0567	Population Size = 18, Crossover rate = 0.67910, Mutation rate = 0.06094, Elitism = 1, Noise scale = 0.01321, Patience = 3	20
ACO	21.2952	Archive Size = 14, Ants = 10, *q* = 0.31414, ξ = 0.96679, σ_min_ = 0.000524	20

#### 4.4.1 PPO training hyperparameter configuration

The PPO agent was trained using the hyperparameter configuration summarized in Table 8. These hyperparameters were adopted during policy training to achieve stable reinforcement learning and efficient policy updates while interacting with the NAMSPSO optimization environment. The learning rate, rollout length, batch size, clipping range, discount factor, Generalized Advantage Estimation (GAE), and entropy coefficient were selected to provide stable convergence during training.

**Table 8:** PPO training hyperparameter configuration.

Hyperparameter	Value
Policy network	MLP Policy
Learning rate	0.0003
Rollout steps	64
Batch size	64
PPO training epochs	4
Discount factor (γ)	0.99
Generalized advantage estimation (λ)	0.95
PPO clipping range	0.20
Entropy coefficient	0.005
Network architecture	MLP (2 × 64, Tanh)
Total training timesteps	15,000

The PPO agent was trained for a total of 15,000 interaction timesteps, after which the learned policy was employed to dynamically select optimization operators during the traffic signal optimization process. The PPO agent employed the default Stable-Baselines3 MlpPolicy, consisting of two fully connected hidden layers with 64 neurons each and Tanh activation functions. Separate policy (actor) and value (critic) networks were used for action selection and value estimation, respectively. The network architecture remained unchanged throughout all training and evaluation experiments. After completion of PPO training, the learned policy was fixed and used during all comparative evaluation experiments without further policy updates.

The PPO agent was trained for 15,000 interaction timesteps, which provided stable policy convergence while maintaining a practical computational cost for repeated SUMO-based optimization. This training duration was selected because additional training beyond 15,000 interaction timesteps produced negligible improvement in the average optimization reward while substantially increasing the computational cost associated with repeated SUMO-based simulations.

### 4.5 Experimental procedure

The proposed PPO-NAMSPSO framework was evaluated under identical simulation conditions to ensure a fair comparison with the benchmark optimization algorithms. The traffic demand, network topology, simulation duration, and traffic signal configuration were kept unchanged throughout all experiments. During each optimization iteration, candidate traffic signal timing plans generated by the optimization algorithm were evaluated within the SUMO simulation environment through the Traffic Control Interface (TraCI), and the resulting traffic performance measurements were returned to the optimizer for updating the particle population. The proposed framework was implemented in Python using Stable-Baselines3 for PPO training, Optuna for hyperparameter optimization, and the SUMO microscopic traffic simulator interfaced through TraCI.

Unlike conventional Particle Swarm Optimization (PSO), which employs a fixed particle update mechanism throughout the optimization process, the proposed framework enables the PPO agent to dynamically select one of six complementary optimization operators according to the current optimization state. This adaptive operator selection allows the optimizer to balance exploration and exploitation during different stages of the search process, thereby generating improved traffic signal timing plans.

Each optimization algorithm was independently trained using five random seeds (42, 52, 62, 72, and 82). The optimized traffic signal timing plans obtained from each training seed were subsequently evaluated over 20 independent SUMO simulation episodes using different evaluation seeds, resulting in a total of 100 evaluation runs for each optimization algorithm. The reported results correspond to the mean and standard deviation computed over these 100 evaluation runs.

To ensure a fair comparison, PSO, GA, ACO, and PPO-NAMSPSO were independently optimized using the Optuna–Tree-Structured Parzen Estimator (TPE) hyperparameter optimization framework under the same optimization budget before comparative evaluation. Subsequently, all algorithms were executed using identical optimization iterations, traffic demand, simulation settings, and evaluation procedures.The experimental evaluation settings adopted for all comparative experiments are summarized in Table 9.

**Table 9:** Experimental evaluation protocol.

Parameter	Value
Training seeds	5 (42, 52, 62, 72, 82)
Evaluation episodes per seed	20
Total evaluation runs	100
Optimization iterations (*K*)	8
Hyperparameter tuning	Optuna–TPE
Compared algorithms	PSO, GA, ACO, PPO-NAMSPSO
Performance statistics	Mean ± Standard deviation

For the comparative evaluation, the maximum number of optimization iterations was fixed at *K* = 8 for all optimization algorithms, including PPO-NAMSPSO, PSO, GA, and ACO, thereby ensuring an identical optimization budget and a fair comparison. During PPO policy training, each training episode consisted of *K* = 8 optimization iterations, whereas the trained policy was evaluated using the same value of *K* = 8 during the final performance comparison.

## 5 Results and analysis

### 5.1 Performance evaluation on the benchmark traffic network

Comparative experiments were conducted to evaluate the effectiveness of the proposed PPO-NAMSPSO framework against four widely used traffic signal optimization methods, namely Fixed-Time Control, Particle Swarm Optimization (PSO), Genetic Algorithm (GA), and Ant Colony Optimization (ACO). All algorithms were evaluated under identical simulation conditions using the same traffic demand, network topology, simulation duration, and optimization budget. Performance was assessed using two primary traffic efficiency metrics: average vehicle waiting time and average vehicle speed. Table 10 presents the comparative performance of the evaluated algorithms on the synthetic benchmark traffic network.

**Table 10:** Performance comparison of traffic signal optimization algorithms on the synthetic benchmark traffic network.

Method	Average waiting time (s) ↓	Improvement of method over PSO (%)	Average speed (m/s) ↑
ACO	113.66	–7.71	8.11
Fixed-time control	127.13	–20.48	7.52
GA	149.43	–41.61	6.83
PSO	105.52	Baseline	8.43
PPO-NAMSPSO (proposed)	96.97	8.10	8.76

As shown in Table 10, the proposed PPO-NAMSPSO framework achieved the lowest average vehicle waiting time among all evaluated methods. Compared with the conventional PSO algorithm, PPO-NAMSPSO reduced the average waiting time by 8.10%, while substantially outperforming Fixed-Time Control, GA, and ACO. This improvement is primarily attributed to the PPO-guided adaptive operator selection mechanism, which dynamically selects the most appropriate optimization operator according to the current optimization state. Unlike conventional PSO, which relies on a fixed particle update strategy throughout the optimization process, the proposed framework balances exploration and exploitation more effectively, enabling the optimizer to discover higher-quality traffic signal timing plans.

Figure 7 illustrates the comparison of the average vehicle waiting time achieved by the evaluated traffic signal optimization algorithms on the synthetic benchmark traffic network. Lower waiting time indicates more efficient traffic signal control and improved traffic flow.

**Figure 7:**
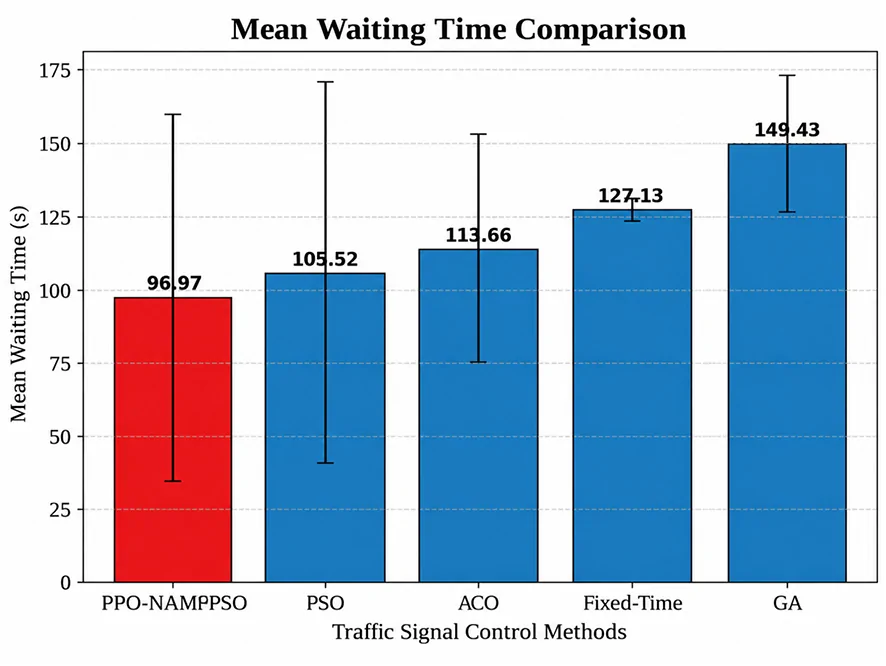
Comparison of average vehicle waiting time on the synthetic benchmark traffic network.

Figure 8 compares the average vehicle speed achieved by the evaluated traffic signal optimization algorithms. Higher average vehicle speed indicates smoother traffic progression, reduced congestion, and fewer unnecessary stops at signalized intersections.

**Figure 8:**
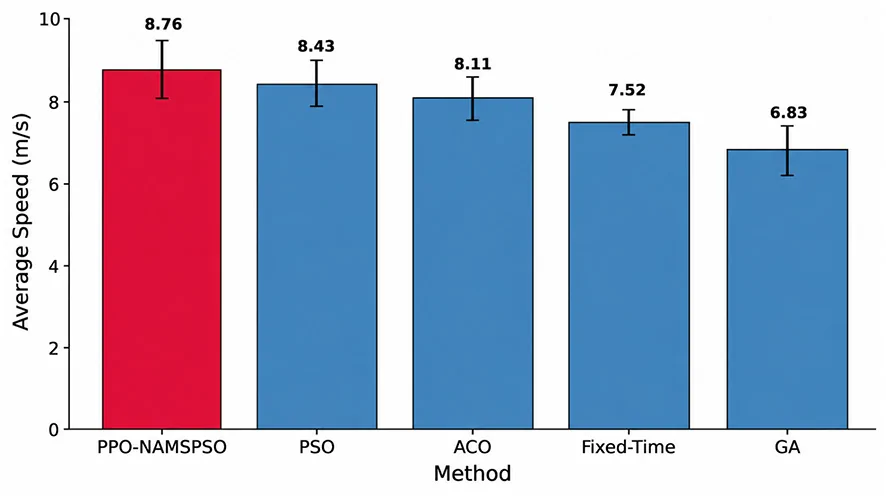
Comparison of average vehicle speed on the synthetic benchmark traffic network.

As shown in Figure 8, PPO-NAMSPSO achieved the highest average vehicle speed among all evaluated methods, reaching 8.76 m/s. The improvement in average speed demonstrates that the proposed adaptive optimization strategy effectively enhances traffic progression by reducing vehicle delays and congestion. This performance gain can be attributed to the PPO-guided adaptive operator selection mechanism, which dynamically balances exploration and exploitation throughout the optimization process, resulting in more efficient traffic signal timing plans than those produced by the benchmark optimization algorithms.

#### 5.1.1 Performance evaluation under high traffic scenario

To evaluate the robustness of the proposed PPO-NAMSPSO framework under severe traffic congestion, comparative experiments were conducted using a high traffic demand scenario. All optimization algorithms were evaluated under identical traffic demand, simulation parameters, and experimental settings to ensure a fair comparison. Performance was assessed using four traffic efficiency metrics, namely average vehicle waiting time, average travel time, congestion ratio, and average vehicle speed, which collectively reflect the effectiveness of each optimization algorithm under heavy traffic conditions. Table 11 summarizes the comparative performance of the evaluated methods.

**Table 11:** Performance comparison under the high traffic scenario.

Method	Waiting time (s) ↓	Travel time (s) ↓	Congestion ratio ↓	Average speed (m/s) ↑
ACO	249.69 ± 61.39	566.22 ± 61.20	0.885 ± 0.056	7.12 ± 0.82
Fixed-time	284.63 ± 6.35	596.25 ± 7.71	0.912 ± 0.005	6.70 ± 0.05
GA	268.06 ± 60.61	580.72 ± 58.53	0.902 ± 0.053	6.99 ± 0.76
PSO	315.91 ± 113.04	627.73 ± 102.39	0.929 ± 0.091	6.64 ± 1.54
PPO-NAMSPSO (proposed)	207.72 ± 47.04	533.38 ± 46.71	0.843 ± 0.047	7.92 ± 0.76

The results presented in Table 11 demonstrate that the proposed PPO-NAMSPSO framework consistently outperformed all comparison algorithms under high traffic demand. Under severe congestion, the optimization landscape becomes considerably more challenging because of the increased interactions among neighboring signalized intersections. The PPO-guided adaptive operator selection mechanism enables the optimizer to dynamically switch between exploration-oriented and exploitation-oriented search operators according to the current optimization state. Consequently, the proposed framework effectively avoids premature convergence and produces higher-quality traffic signal timing plans than conventional PSO and the other benchmark optimization algorithms.

Figure 9 compares the average vehicle waiting time achieved by the evaluated traffic signal optimization algorithms under the high traffic demand scenario. Lower waiting time indicates more effective traffic signal coordination and improved traffic flow during severe congestion.

**Figure 9:**
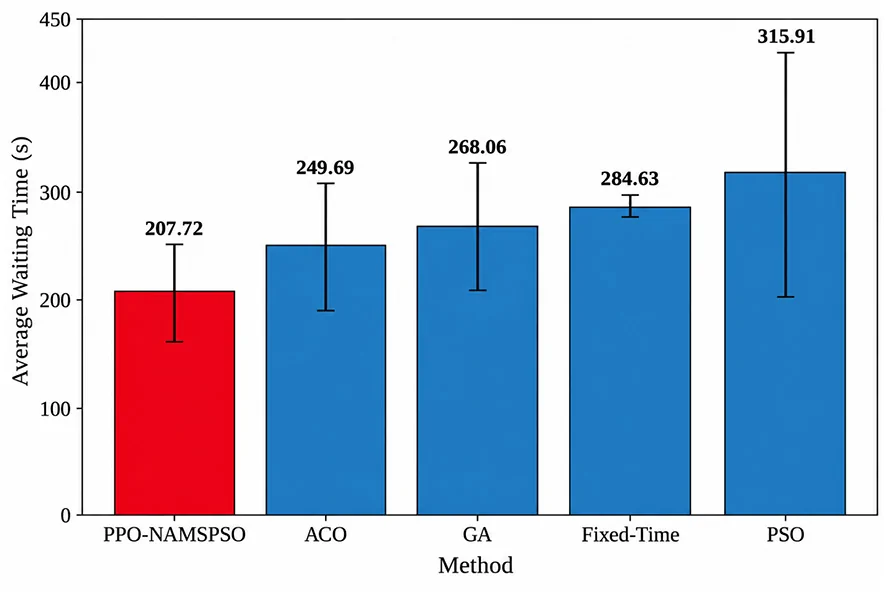
Comparison of average vehicle waiting time under the high traffic demand scenario.

Figure 9 visually confirms the superior waiting-time performance reported in Table 11. The proposed PPO-NAMSPSO framework consistently maintained the lowest vehicle delay under heavy traffic conditions because the adaptive operator selection mechanism effectively balanced exploration and exploitation, enabling the optimizer to avoid premature convergence and identify more efficient traffic signal timing plans.

Figure 10 compares the average vehicle speed achieved by the evaluated optimization algorithms under the high traffic demand scenario. Higher average vehicle speed indicates smoother traffic progression and reduced congestion throughout the traffic network.

**Figure 10:**
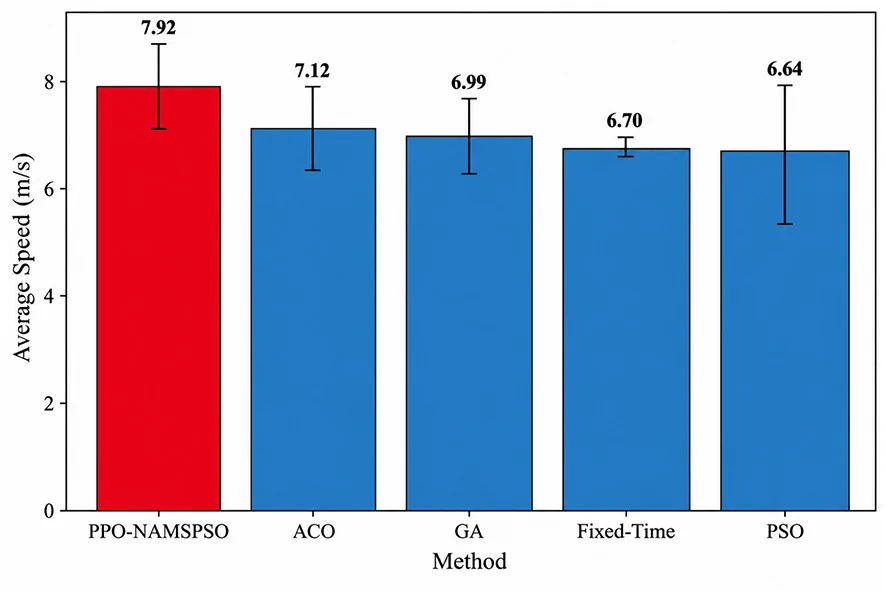
Comparison of average vehicle speed under the high traffic demand scenario.

As shown in Figure 10, PPO-NAMSPSO achieved the highest average vehicle speed among all evaluated methods, reaching 7.92 ± 0.76 m/s. The simultaneous reduction in waiting time, travel time, and congestion ratio, together with the increase in average vehicle speed, demonstrates the robustness of the proposed framework under severe traffic congestion. These results indicate that the PPO-guided adaptive operator selection strategy enables the optimizer to maintain efficient traffic flow even under highly congested traffic conditions.

### 5.2 Performance evaluation on the OSM-based urban traffic network

To evaluate the scalability and practical applicability of the proposed PPO-NAMSPSO framework, comparative experiments were further conducted on a realistic OpenStreetMap (OSM)-based urban traffic network comprising 25 signalized intersections. Unlike the synthetic benchmark network, the OSM network contains irregular road geometries, heterogeneous traffic movements, and more complex interactions among neighboring intersections, thereby providing a considerably more challenging optimization environment. All comparison algorithms were evaluated under identical traffic demand, simulation parameters, and optimization settings to ensure a fair and unbiased performance comparison.

Table 12 summarizes the comparative performance of the evaluated traffic signal optimization algorithms on the OSM-based urban traffic network.

**Table 12:** Performance comparison of traffic signal optimization algorithms on the OSM-based urban traffic network.

Method	Average waiting time (s) ↓	Standard deviation (s)	Improvement over PSO (%)	Average speed (m/s) ↑
ACO	39.79	12.83	18.59	13.07
Fixed-time control	40.52	2.82	17.11	12.92
GA	40.21	11.51	17.74	13.04
PSO	48.88	17.42	Baseline	12.94
PPO-NAMSPSO (proposed)	33.07	3.54	32.35	13.08

As shown in Table 12, the proposed PPO-NAMSPSO framework achieved the best overall performance on the realistic OSM-based urban traffic network. The proposed framework reduced the average vehicle waiting time to 33.07 s, whereas the conventional PSO algorithm recorded an average waiting time of 48.88 s, corresponding to a 32.35% reduction. Furthermore, PPO-NAMSPSO consistently outperformed Fixed-Time Control, GA, and ACO in maintaining efficient traffic flow under complex urban traffic conditions.

In addition to achieving the lowest average waiting time, the proposed framework exhibited substantially lower performance variability than conventional PSO. The standard deviation of vehicle waiting time decreased from 17.42 s for PSO to 3.54 s for PPO-NAMSPSO, indicating significantly more stable optimization performance across repeated simulation runs. These results demonstrate that the proposed adaptive optimization strategy not only improves traffic efficiency but also enhances the robustness and consistency of the optimization process.

Figure 11 compares the average vehicle waiting time achieved by the evaluated traffic signal optimization algorithms on the OSM-based urban traffic network. Lower waiting time indicates more effective traffic signal coordination and improved traffic flow in a realistic urban environment.

**Figure 11:**
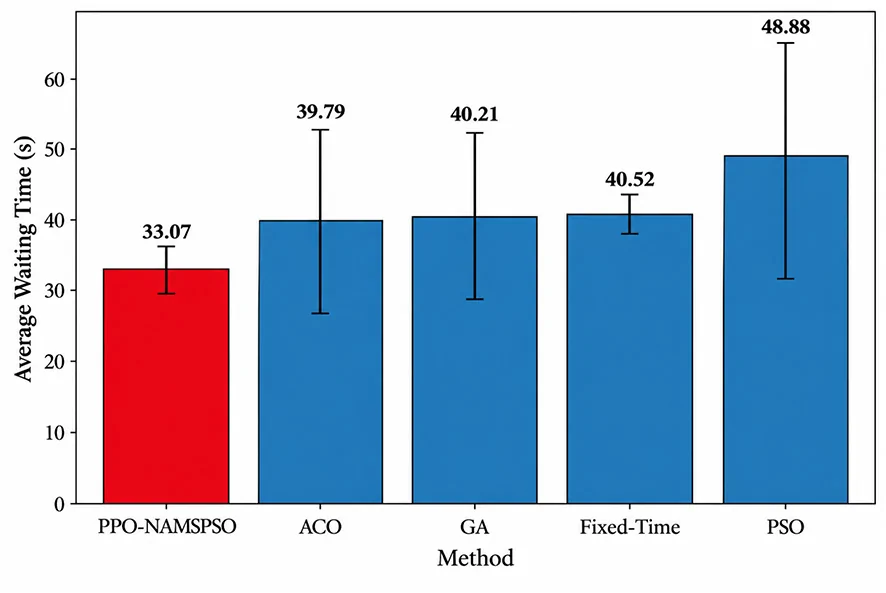
Comparison of average vehicle waiting time on the OSM-based urban traffic network.

Figure 11 visually confirms the substantial waiting-time reduction achieved by the proposed PPO-NAMSPSO framework. Compared with the benchmark optimization algorithms, the performance improvement is more pronounced on the realistic OSM network, demonstrating the scalability of the proposed framework to larger and more complex traffic optimization problems.

Figure 12 compares the average vehicle speed obtained by the evaluated optimization algorithms on the OSM-based urban traffic network. Higher average vehicle speed indicates smoother traffic progression and reduced congestion throughout the urban road network.

**Figure 12:**
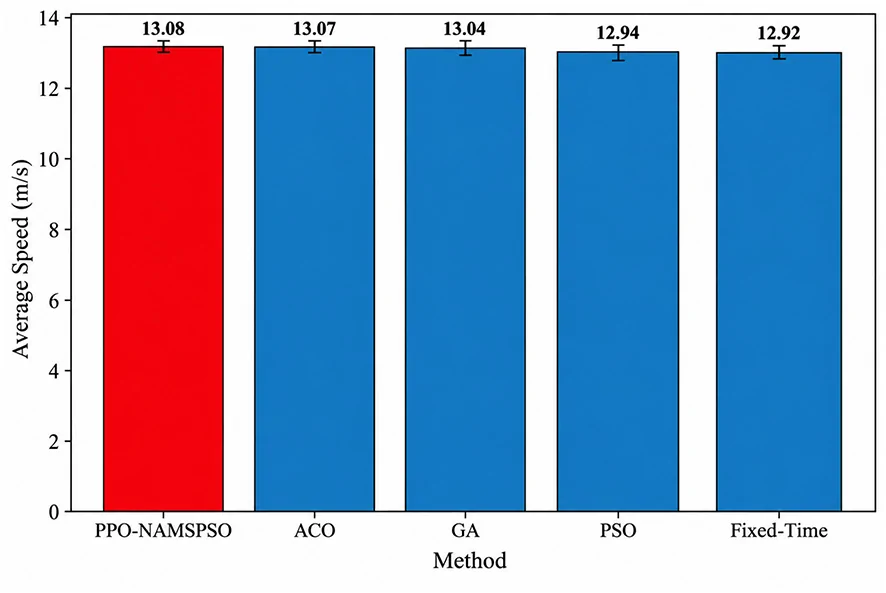
Comparison of average vehicle speed on the OSM-based urban traffic network.

As shown in Figure 12, the proposed PPO-NAMSPSO framework achieved the highest average vehicle speed of 13.08 m/s among all evaluated methods. Conventional PSO achieved an average speed of 12.94 m/s, while ACO, GA, and Fixed-Time Control achieved 13.07 m/s, 13.04 m/s, and 12.92 m/s, respectively. The increase in average vehicle speed indicates that the proposed framework maintains smoother traffic progression while simultaneously reducing vehicle waiting time.

Overall, the experimental results obtained on the OSM-based urban traffic network demonstrate that the proposed PPO-NAMSPSO framework scales effectively from the synthetic benchmark network to realistic urban traffic environments. The significant reduction in average waiting time, lower performance variability, and improved average vehicle speed confirm the robustness, scalability, and practical applicability of the proposed framework for large-scale adaptive traffic signal optimization. The greater performance improvement observed on the OSM network is primarily attributed to its larger and more complex search space, where the PPO-guided adaptive operator selection mechanism provides greater benefits by dynamically balancing exploration and exploitation throughout the optimization process.

### 5.3 Analysis of PPO-based adaptive operator selection

To better understand the behavior of the proposed PPO-NAMSPSO framework, the optimization operator selected by the PPO agent was recorded throughout the optimization process. Table 13 summarizes the frequency with which each optimization operator was selected during all optimization iterations.

**Table 13:** Frequency of optimization operator selection by the PPO agent.

PPO action	Optimization operator	Number of selections	Selection frequency (%)
0	Standard PSO update	626	17.60
1	Novelty-guided exploration	589	16.56
2	Elite-guided update	733	20.61
3	Mutation-based search	579	16.28
4	Restart strategy	483	13.58
5	Local search	547	15.38

As shown in Table 13, the learned PPO policy employed all six complementary optimization operators rather than relying on a single fixed particle update strategy. Among the available operators, the Elite-Guided Update was selected most frequently, accounting for 20.61% of all operator selections. This indicates that exploiting high-quality candidate solutions plays an important role during the optimization process. The Standard PSO Update, Novelty-Guided Exploration, Mutation-Based Search, Restart Strategy, and Local Search operators were also selected with comparable frequencies, demonstrating that each operator contributed to the optimization process under different search conditions.

The operator selection frequencies reported in Table 13 were accumulated over all optimization iterations performed during the high-traffic experiments on both the synthetic benchmark network and the realistic OSM-based urban traffic network. The relatively balanced distribution of operator selections indicates that the PPO agent learned to adapt its search strategy according to the current optimization state rather than repeatedly applying a single optimization operator throughout the search process.

This adaptive operator selection behavior demonstrates that the reinforcement learning agent successfully balanced global exploration and local exploitation during optimization. Exploration-oriented operators, including Novelty-Guided Exploration, Mutation-Based Search, and the Restart Strategy, were selected to maintain population diversity and prevent premature convergence. In contrast, exploitation-oriented operators, such as the Elite-Guided Update and Local Search, were employed to refine promising candidate solutions and accelerate convergence toward high-quality traffic signal timing plans.

Overall, the analysis confirms that the proposed PPO-guided adaptive operator selection mechanism effectively controlled the optimization behavior of the NAMSPSO algorithm by dynamically selecting the most appropriate search operator according to the current optimization state. This adaptive decision-making capability directly supports the primary contribution of the proposed framework and explains the consistent performance improvements observed over the benchmark optimization algorithms in both the synthetic benchmark and realistic OSM-based traffic networks.

### 5.4 Statistical validation of the OSM experimental results

To assess whether the observed performance differences among the evaluated traffic signal optimization methods on the OpenStreetMap (OSM)-based urban traffic network were statistically significant and unlikely to arise from random variation across repeated runs, statistical analyses were performed using evaluation results obtained from 100 repeated runs with different random seeds for each evaluated method. Both parametric and non-parametric statistical tests were considered to provide a comprehensive assessment of the observed performance differences. Overall differences among the evaluated methods were examined using one-way analysis of variance (ANOVA) and the Kruskal–Wallis test. Pairwise comparisons between the proposed PPO-NAMSPSO framework and the benchmark methods were further performed using Welch's *t*-test with Holm correction for multiple comparisons. Effect sizes were also reported to quantify the practical magnitude of the observed differences. Statistical significance was assessed at the α = 0.05 level.

A one-way analysis of variance (ANOVA) was first performed to examine whether significant differences existed among the evaluated methods. For average vehicle waiting time, the ANOVA result indicated a statistically significant difference among the methods (*F* = 25.375, *p* < 0.001). The non-parametric Kruskal–Wallis test was additionally applied as a distribution-independent overall comparison and also confirmed a statistically significant difference among the evaluated methods (*H* = 89.339, *p* < 0.001).

To further investigate the differences between the proposed PPO-NAMSPSO framework and the benchmark methods, pairwise Welch's *t*-tests were conducted, with Holm correction applied to account for multiple comparisons. The PPO-NAMSPSO framework showed statistically significant differences in average waiting time compared with Fixed-Time Control ($p_{\text{Holm}}=2.51\times 10^{-37}$), ACO ($p_{\text{Holm}}=1.17\times 10^{-5}$), GA ($p_{\text{Holm}}=2.52\times 10^{-7}$), and PSO ($p_{\text{Holm}}=1.41\times 10^{-13}$). The corresponding effect sizes (Cohen's *d*) were large for the comparisons with Fixed-Time Control (*d* = 2.33), GA (*d* = 0.84), and PSO (*d* = 1.26), and medium for the comparison with ACO (*d* = 0.71). These results indicate that the observed reduction in average vehicle waiting time achieved by PPO-NAMSPSO is not only statistically significant but also associated with practically meaningful effect sizes.

The mean waiting time obtained by PPO-NAMSPSO was 33.07 s, compared with 40.52 s for Fixed-Time Control, 39.79 s for ACO, 40.21 s for GA, and 48.88 s for PSO. To explicitly distinguish these comparisons from the method-over-PSO percentages reported in Table 11, the following percentages represent the improvement of PPO-NAMSPSO over each respective benchmark method. PPO-NAMSPSO achieved waiting-time reductions of approximately 18.40%, 16.90%, 17.76%, and 32.35% relative to Fixed-Time Control, ACO, GA, and PSO, respectively.

Overall, the statistical analysis provides evidence that PPO-NAMSPSO achieves significantly lower average vehicle waiting time than all evaluated benchmark methods on the OSM-based urban traffic network, with effect sizes ranging from medium to large. The statistical results are summarized in Table 14.

**Table 14:** Statistical validation of average vehicle waiting time on the OSM-based urban traffic network.

Statistical test	Comparison	Result	Interpretation
One-way ANOVA	All evaluated methods	*F* = 25.375, *p* < 0.001	A statistically significant difference exists among the evaluated methods.
Kruskal–Wallis	All evaluated methods	*H* = 89.339, *p* < 0.001	The overall difference among the methods remains statistically significant under the non-parametric test.
Welch's *t*-test + Holm	PPO-NAMSPSO vs. Fixed-Time	$p_{\text{Holm}}=2.51\times 10^{-37}$, *d* = 2.33	PPO-NAMSPSO significantly outperforms Fixed-Time Control with a large effect size.
Welch's *t*-test + Holm	PPO-NAMSPSO vs. ACO	$p_{\text{Holm}}=1.17\times 10^{-5}$, *d* = 0.71	PPO-NAMSPSO significantly outperforms ACO with a medium effect size.
Welch's *t*-test + Holm	PPO-NAMSPSO vs. GA	$p_{\text{Holm}}=2.52\times 10^{-7}$, *d* = 0.84	PPO-NAMSPSO significantly outperforms GA with a large effect size.
Welch's *t*-test + Holm	PPO-NAMSPSO vs. PSO	$p_{\text{Holm}}=1.41\times 10^{-13}$, *d* = 1.26	PPO-NAMSPSO significantly outperforms PSO with a large effect size.

*F* denotes the one-way ANOVA statistic, *H* denotes the Kruskal–Wallis statistic, *p*_Holm_ denotes the Holm-adjusted *p*-value, *d* denotes Cohen's effect size, and statistical significance was assessed at α = 0.05.

### 5.5 Ablation study

To quantify the contribution of the reinforcement learning component, an ablation study was conducted under the high traffic demand scenario by comparing the complete PPO-NAMSPSO framework with a baseline variant operating without the PPO-guided adaptive operator selection mechanism. The high traffic scenario was selected because it represents the most challenging traffic condition, where the benefits of adaptive optimization strategies are expected to be most pronounced. Table 15 summarizes the comparative performance of the two framework variants.

**Table 15:** Ablation study evaluating the contribution of the PPO-based adaptive operator selection module.

Method	Mean waiting time (s) ↓	Standard deviation (s)	Mean optimization time (s)	Improvement (%) ↑
Without PPO	64.91	10.29	43.74	Baseline
PPO-NAMSPSO (proposed)	60.38	9.55	43.79	6.98

As shown in Table 15, the proposed PPO-NAMSPSO framework achieved superior optimization performance compared with the variant operating without the PPO-guided adaptive operator selection mechanism. The complete framework reduced the mean vehicle waiting time from 64.91 s to 60.38 s, corresponding to a 6.98% improvement in traffic signal optimization performance. This result demonstrates that the reinforcement learning component contributes directly to improving the quality of the optimized traffic signal timing plans.

In addition to improving traffic efficiency, the proposed framework also exhibited greater optimization stability. The standard deviation of the waiting time decreased from 10.29 s to 9.55 s, indicating more consistent optimization performance across repeated simulation runs. The reduction in performance variability suggests that the PPO-guided adaptive operator selection mechanism enables the optimizer to avoid premature convergence and maintain a more balanced search throughout the optimization process.

Interestingly, the computational cost of both framework variants remained nearly identical. The mean optimization time increased only marginally from 43.74 s to 43.79 s, indicating that the integration of the PPO agent introduces negligible computational overhead while providing a measurable improvement in optimization quality. Therefore, the observed performance gains are primarily attributed to intelligent adaptive strategy selection rather than additional computational effort.

Overall, the ablation study confirms that the PPO-guided adaptive operator selection mechanism is an essential component of the proposed PPO-NAMSPSO framework. By dynamically selecting suitable optimization operators according to the current optimization state, the reinforcement learning agent consistently generates higher-quality traffic signal timing plans, reduces vehicle waiting time, improves optimization stability, and enhances the overall effectiveness of adaptive traffic signal optimization.

### 5.6 Overall discussion

The experimental results consistently demonstrate that the proposed PPO-NAMSPSO framework significantly improves adaptive traffic signal optimization by integrating PPO-guided adaptive operator selection with the Novelty-Aware Multi-Strategy Particle Swarm Optimization algorithm. Across both the synthetic benchmark network and the realistic OSM-based urban traffic network, the proposed framework consistently achieved lower vehicle waiting times, higher average vehicle speeds, and more stable optimization performance than the benchmark methods, including Fixed-Time Control, PSO, GA, and ACO.

The superior performance of PPO-NAMSPSO can be primarily attributed to its adaptive optimization strategy. Unlike conventional PSO, which applies a fixed particle update mechanism throughout the optimization process, the proposed framework dynamically selects one of six complementary optimization operators according to the current optimization state. This adaptive decision-making capability enables the optimizer to balance global exploration and local exploitation more effectively, thereby avoiding premature convergence while maintaining continuous search diversity. Consequently, the optimizer is able to identify higher-quality traffic signal timing plans under varying traffic conditions.

The ablation study further confirmed the importance of the reinforcement learning component. Removing the PPO-guided adaptive operator selection mechanism resulted in higher vehicle waiting times and greater performance variability, demonstrating that the reinforcement learning agent contributes directly to optimization quality rather than simply increasing computational effort. Furthermore, the negligible increase in optimization time indicates that the proposed adaptive strategy provides substantial performance improvements with minimal computational overhead.

The statistical analyses performed on the OSM-based urban traffic network further strengthen the validity of the proposed framework. The one-way ANOVA and the non-parametric Kruskal–Wallis test confirmed a statistically significant overall difference among the evaluated methods, and the pairwise Welch's t-tests with Holm correction confirmed that PPO–NAMSPSO's improvements over each individual benchmark algorithm were statistically significant, with effect sizes ranging from medium (ACO) to large (Fixed-Time Control, GA, PSO). These findings demonstrate that the performance gains achieved by PPO–NAMSPSO are reliable and cannot be attributed to random variations in the optimization process.

From the perspective of Intelligent Transportation Systems (ITS), the proposed framework has significant practical implications for adaptive urban traffic management. By minimizing vehicle waiting time, improving traffic progression, and maintaining efficient traffic flow under different traffic demand conditions, PPO-NAMSPSO has the potential to alleviate urban congestion, reduce travel delays, and improve the operational efficiency of signalized road networks. The successful evaluation on both benchmark and realistic OSM-based traffic networks further demonstrates the scalability and applicability of the proposed framework to complex urban transportation environments.

Although the proposed framework achieved promising results, several opportunities remain for future research. The current study was conducted using microscopic traffic simulation under predefined traffic demand scenarios. Future work will focus on validating the proposed framework using real-world traffic datasets, integrating connected and autonomous vehicle information, extending the optimization framework to coordinated corridor and city-scale traffic networks, and investigating multi-objective optimization strategies that simultaneously consider traffic efficiency, fuel consumption, emissions, and environmental sustainability.

Overall, the comprehensive experimental evaluation demonstrates that PPO-NAMSPSO is a robust, scalable, and computationally efficient optimization framework for adaptive traffic signal control. The combination of reinforcement learning and novelty-aware multi-strategy optimization provides a promising direction for next-generation intelligent traffic management systems operating in increasingly complex urban environments.

## 6 Conclusion and future work

This paper presented a PPO-NAMSPSO framework for adaptive traffic signal optimization and evaluated its performance on both a synthetic benchmark traffic network and a realistic OpenStreetMap (OSM)-based urban traffic network. The proposed framework integrates reinforcement learning with a novelty-aware multi-strategy particle swarm optimization algorithm, enabling the optimizer to dynamically select the most appropriate search operator according to the current optimization state.

The experimental results demonstrated that PPO–NAMSPSO consistently outperformed the benchmark traffic signal optimization methods, including Fixed-Time Control, Particle Swarm Optimization (PSO), Genetic Algorithm (GA), and Ant Colony Optimization (ACO). Across different traffic scenarios, the proposed framework achieved lower average vehicle waiting time, shorter travel time, reduced congestion ratio, and higher average vehicle speed than the benchmark algorithms. Furthermore, statistical validation using one-way ANOVA, the Kruskal–Wallis test, and pairwise Welch's t-tests with Holm correction confirmed that the observed performance improvements were statistically significant. The ablation study further demonstrated that the PPO-guided adaptive operator selection mechanism is a key contributor to the optimization performance, improving traffic signal timing quality while introducing only negligible computational overhead.

Although the proposed framework demonstrated promising performance, several limitations remain. The current implementation was evaluated using microscopic SUMO simulations on both a synthetic benchmark network and an OpenStreetMap (OSM)-derived urban road network; however, the traffic demand used in the experiments was synthetically generated under predefined traffic demand patterns rather than obtained from real-world traffic measurements. Therefore, although the OSM-based network provides realistic road geometry and intersection characteristics, the experiments do not reproduce field-observed traffic demand distributions. In addition, the current implementation assumes reliable traffic state information. Practical deployment may require the framework to operate under noisy sensor measurements, communication delays, incomplete observations, and dynamically changing traffic conditions that were not explicitly considered in this study. The current optimization objective primarily focuses on improving traffic efficiency through reduced vehicle waiting time and enhanced traffic progression.

Future work will focus on extending the proposed framework to large-scale city-wide traffic networks with coordinated multi-intersection traffic signal control. Further research will investigate multi-agent reinforcement learning architectures, online learning under non-stationary traffic conditions, and integration with connected and autonomous vehicle environments. Moreover, incorporating additional optimization objectives, including fuel consumption, vehicle emissions, emergency vehicle priority, and pedestrian safety, will further enhance the applicability of the proposed framework for intelligent transportation systems. Finally, validation using real-world traffic datasets and hardware-in-the-loop experiments will be pursued to further assess the practicality, robustness, and deployment potential of the proposed PPO-NAMSPSO framework in real-world urban traffic management systems.

## Data Availability

The raw data supporting the conclusions of this article will be made available by the authors, without undue reservation.
